# A CD57^+^CD8^+^ T cell subset links T cell cytotoxicity to fibrotic lung disease in systemic sclerosis

**DOI:** 10.1172/JCI194288

**Published:** 2026-02-17

**Authors:** Takanori Sasaki, Ye Cao, John M. Sowerby, Kazuhiko Higashioka, Kathryne E. Marks, Mehreen Elahee, Mari Kamiya, Paul F. Dellaripa, Richard I. Ainsworth, Kimberly E. Taylor, Nunzio Bottini, Paul Wolters, Edy Y. Kim, Francesco Boin, Deepak A. Rao

**Affiliations:** 1 Division of Rheumatology, Inflammation, and Immunity, Department of Medicine, Brigham and Women’s Hospital (BWH) and Harvard Medical School, Boston, Massachusetts, USA.; 2 Division of Rheumatology, Department of Internal Medicine, Keio University School of Medicine, Tokyo, Japan.; 3 Division of Pulmonary and Critical Care Medicine, Department of Medicine, BWH and Harvard Medical School, Boston, Massachusetts, USA.; 4 Department of Medicine, Division of Rheumatology, and; 5 Kao Autoimmunity Institute, Cedars-Sinai Medical Center, Los Angeles, California, USA.; 6 Division of Rheumatology, Department of Medicine, and; 7 Division of Pulmonary, Critical Care, Allergy and Sleep, Department of Medicine, UCSF, San Francisco, California, USA.

**Keywords:** Autoimmunity, Immunology, T cells

## Abstract

Interstitial lung disease (ILD) is a major cause of morbidity and mortality in systemic sclerosis (SSc); however, the immunopathologic mechanisms driving lung disease in SSc are unclear. T cells have been implicated as a likely driver of lung injury in SSc. Here, we evaluated T cells in the blood of patients with SSc-ILD and identified a specific population of cytotoxic CD8^+^ T cells that was expanded in patients with SSc-ILD. Cytotoxic effector memory CD8^+^ T cells marked by CD57 expression were preferentially expanded in patients with SSc-ILD compared with patients with SSc but no ILD and control individuals and showed prominent clonal expansion. These CD57^+^ T effector memory (Tem) cells differed from T effector memory cells reexpressing CD45RA (Temra) transcriptomically and functionally, with cytotoxic function that was enhanced by CD155 engagement of the costimulatory receptor CD226. We performed immunostaining of lung tissue samples obtained from independent patients with SSc-ILD (biopsy or explant) and confirmed the presence of CD57^+^ Tem cells. In parallel, we analyzed publicly available lung scRNA-seq datasets from multiple ILD cohorts and identified endothelial cells as a likely source of CD155 for the activation of CD57^+^ cytotoxic T cells. Together, the results implicate a CD57^+^ cytotoxic CD8^+^ T cell population as a potential mediator of lung injury in SSc-ILD.

## Abstract 2

CD57+ cytotoxic effector memory CD8 T cells may drive lung damage in systemic sclerosis-related lung disease, with endothelial cells potentially enhancing their activity through the CD155-CD226 pathway.

## Introduction

Systemic sclerosis (SSc) is a systemic autoimmune disease characterized by hardening of skin, fibrosis, and vascular endothelial damage (1). The disease can affect vital organs such as lungs, heart, and kidneys. Interstitial lung disease (ILD) is a frequent complication, occurring in approximately 40%–75% of patients, and is associated with a poor prognosis (1). Mycophenolate mofetil (2), cyclophosphamide (3, 4), rituximab (5), tocilizumab (6), and nintedanib (7) have shown some benefit in the treatment of SSc-ILD (8–10); however, the efficacy of these treatments remains limited, and SSc-ILD is still a leading cause of SSc-related mortality (11), underscoring the need for novel therapeutic approaches.

SSc-ILD pathology is characterized by irreversible and progressive pulmonary fibrosis. Early detection can improve the prognosis; thus, current guidelines recommend screening for ILD using pulmonary function tests (PFTs), high-resolution CT (HRCT), and Scl-70 antibody testing, as well as monitoring ILD progression by PFT and HRCT after initiating SSc-ILD treatment (8–10). These metrics primarily evaluate pulmonary function decline and the extent or progression of lung fibrosis after it has already occurred but do not quantify the magnitude of the ongoing immune-mediated injury. Delineation of such immunologic drivers in the peripheral blood of patients with SSc-ILD could aid the prompt identification of patients at risk for more severe and progressive SSc-ILD, allow measurement of the extent of the pathologic immune activation, and define with greater precision the efficacy of the ongoing treatments in suppressing potential immune-mediated injury.

Several studies have implicated T cells as drivers of tissue injury in SSc. Both CD4^+^ and CD8^+^ cytotoxic T lymphocytes (CTLs) accumulate within the skin of patients with SSc and induce apoptotic death in dermal endothelial cells, potentially leading to tissue damage and fibrosis (12). A programmed death 1–high (PD-1^hi^) cytotoxic CD4^+^ T cell population is also expanded in the circulation of patients with SSc-ILD (13). In addition, IL-13^+^CD8^+^ T cells are increased in the blood and skin of patients with SSc, and their Th2-like functions contribute to skin fibrosis (14, 15). Moreover, a recent single-cell RNA-seq (scRNA-seq) study using SSc-ILD lung samples revealed an increase in CD8^+^ tissue-resident memory T cells (16). However, the features of pathologic T cells that are most prominently activated in patients with SSc-ILD remain incompletely defined. In particular, it is unclear which cytotoxic T cell subsets contribute to ILD pathogenesis, what surface markers define these cells, and how their activation is regulated in the fibrotic lung environment. A deeper understanding of these aspects is essential for identifying immune pathways that could serve as targets for intervention. In this study, we generated extensive mass cytometry and scRNA-seq data on circulating lymphocytes from patients with SSc and sought to identify T cell phenotypes associated with the presence and severity of ILD. We report for the first time to our knowledge that CD57^+^ effector memory (CD57^+^ Tem) CD8^+^ T cells were increased in patients with SSc-ILD and associated with ILD severity. This T cell subset showed oligoclonality and accumulated within affected areas of fibrotic lungs. Their cytotoxic function was enhanced by the activation of surface CD226, potentially by interaction with CD155 expressed on endothelial cells. Together, these findings highlight a cytotoxic pathway mediated by CD57^+^ Tem CD8^+^ T cells in SSc patients with ILD, with CD155 on endothelial cells being a potential initiator of their activation and function.

## Results

### CD57^+^ Tem cells are expanded and associated with disease severity in SSc-ILD.

**Figure 1:**
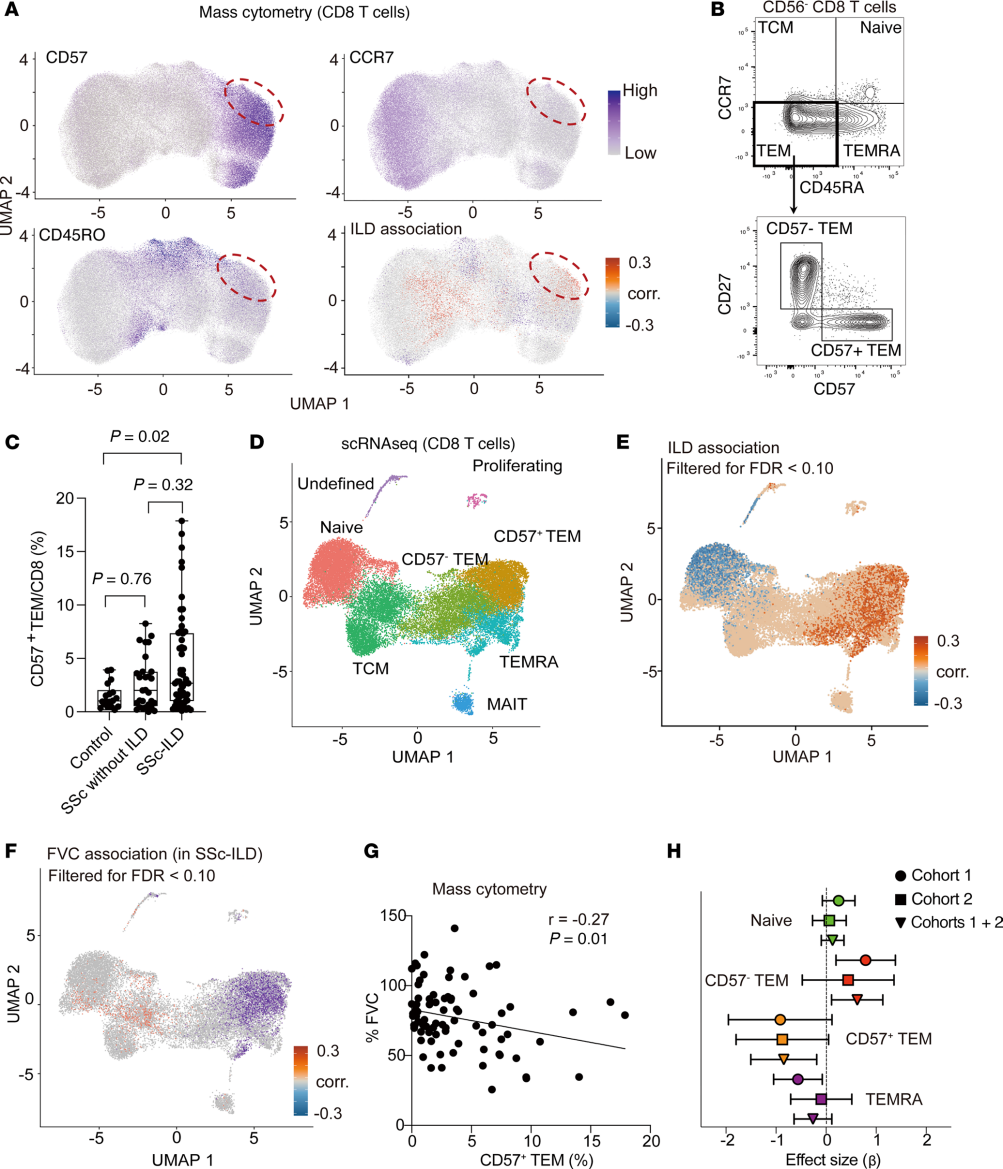
CD57^+^ Tem cells are expanded in patients with SSc-ILD. (**A**) CNA of CD8^+^ T cells from HCs and patients with SSc-ILD, adjusting for age and sex. Red dashed areas indicate cell neighborhoods that were enriched in patients with SSc-ILD. (**B**) Flow cytometric detection of CD57 and CD27 expression in CD8^+^ Tem cells. (**C**) Quantification of CD57^+^CD27^–^CCR7^–^CD45RO CD56^–^ cells among CD8^+^ T cells (HCs: *n* = 18, SSc–non-ILDs: *n* = 29, SSc-ILDs: *n* = 53). *P* values were determined by Kruskal-Wallis test with Dunn’s multiple-comparison test. (**D**) UMAP of CD8^+^ T cell clusters in the scRNA-seq dataset (SSc–non-ILDs: *n* = 20, SSc-ILDs: *n* = 38). (**E**) CNA of CD8^+^ T cells from SSc–non-ILD and SSc-ILD patients, adjusting for age and sex. Red indicates the cell neighborhood enriched in patients with SSc-ILD. (**F**) CNA to assess the correlation with FVC, adjusting for age and sex. (**G**) Correlation analysis between CD57^+^ Tem cells and FVC in patients with SSc who underwent a pulmonary function test (*n* = 79). Spearman’s statistic is shown. (**H**) Effect sizes of the association between CD8^+^ T cell subsets and FVC using a linear mixed-effects model (fixed effects: age and sex, random effect: cohort). Data show the mean ± 95% CI. Box plots represent median and interquartile range (25th-75th percentiles), with whiskers indicating minimum and maximum values.

To identify disease-associated changes in circulating lymphocytes in patients with SSc-ILD, we evaluated PBMCs from a cross-sectional cohort including 53 patients with SSc-ILD, 29 patients with SSc with no ILD, and 18 healthy control (HC) patients without known autoimmune disease. In this cohort (cohort 1), the patients were mostly middle-aged (56 ± 12 years) females (89.0%), predominantly White (59.8%), and had variable skin involvement (diffuse cutaneous SSc in 39.0%). Detailed sociodemographic and clinical information is reported in Table 1. We performed mass cytometry to analyze PBMCs from these patients and focused our analyses on CD8^+^ T cells, given previous evidence supporting significant roles for this cell subset in SSc and ILD pathogenesis (12, 14–16). To identify CD8^+^ T cell phenotypes associated with SSc-ILD, we utilized covarying neighborhood analysis (CNA) (17), a cluster-free method to identify disease-associated cell phenotypes in an unbiased manner. CNA comparing CD8^+^ T cells from patients with SSc-ILD and controls identified a region of T cells significantly increased in patients with SSc-ILD (FDR <0.01, Figure 1A). This region contained cells marked by the expression of CD57 and CD45RO and lacking CCR7, indicating a CD57^+^ effector memory (CD57^+^ Tem) phenotype. This population also exhibited high expression of T-bet and CX3CR1, two markers associated with CTLs, as well as the activation markers CD38 and HLA-DR, and low expression of CD27 and CD56 (Supplemental Figure 1; supplemental material available online with this article; https://doi.org/10.1172/JCI194288DS1). Validation staining on a subset of samples by flow cytometry confirmed that CD57 and CD27 demonstrated an inverse expression pattern on CD8^+^ Tem cells (Figure 1B and Supplemental Figure 2A).

Manual gating of CD8^+^ T cells in the mass cytometry dataset confirmed that CD57^+^CD27^–^CCR7^–^CD45RO^+^CD8^+^ T cells were significantly increased in patients with SSc-ILD compared with controls, but not in SSc patients without ILD (*P* = 0.02; control vs. SSc-ILD, mean: control 1.5%, SSc without ILD 2.7%, SSc-ILD 4.5%, Figure 1C). To confirm the expression of cytotoxic proteins in CD57^+^ Tem cells, we performed intracellular staining for granzymes using samples from 10 controls, 5 patients with SSc without ILD, and 5 patients with SSc-ILD. Flow cytometric analysis validated that almost 90% of CD57^+^ Tem cells expressed granzyme B. CD57^+^ Tem cells showed levels of granzyme B comparable to those in Temra cells, a well-known granzyme B^+^ CD8^+^ T cell population (Supplemental Figure 2, B and C). The levels of granzyme B in CD57^+^ Tem cells were similar across controls, SSc-ILD, and SSc-without-ILD groups, suggesting that high granzyme B expression is an intrinsic feature of CD57^+^ Tem cells.

To extend the characterization of expanded CD8^+^ T cells in patients with SSc-ILD, we analyzed PBMCs from the same cohort of patients with SSc by scRNA-seq and applied CNA to the CD8^+^ T cells in the scRNA-seq data. CNA revealed the enrichment of a similar cytotoxic CD8^+^ T cell population in patients with SSc-ILD in the scRNA-seq dataset, highlighting a region of CD8^+^ T cells with high expression of *GZMB* and *HNRNPLL*, a regulator of CD45RA splicing upregulated in CD45RO^+^ cells (Figure 1, D and E, and Supplemental Figure 3A). Although *B3GAT1* (encoding CD57) expression was not well detected in the RNA-seq data, the identified cell population lacked *CD27* expression, indicating that a similar CTL population was identified in both the mass cytometry and scRNA-seq analyses (Figure 1, D and E, and Supplemental Figure 3A).

To identify cell phenotypes associated with ILD severity, we performed CNA to test for cell regions associated with forced vital capacity (FVC) among the patients with SSc-ILD. This analysis again highlighted a region containing likely CD57^+^ Tem cells associated with SSc-ILD severity, with a negative correlation between cells in this region and FVC (Figure 1F). Manual gating of mass cytometric data confirmed a significant negative association between CD57^+^ Tem cells and FVC (Figure 1G). To further validate the association between CD57^+^ Tem cells and SSc-ILD severity, we performed flow cytometry to quantify CD57^+^ Tem cells in samples from an independent cohort of 42 patients with SSc-ILD (cohort 2) with clinical features similar to those of the initial cohort (Table 1). A linear mixed-effects model assessing cytometric data from cohorts 1 and 2 confirmed a consistent negative association between CD57^+^ Tem cells and FVC across both cohorts (Figure 1H, Cohorts 1 + 2, *P* = 0.009). In contrast, CNA of cohort 1 and flow cytometric analysis of cohort 2 showed no association between CD57^+^ Tem cells and the extent of skin involvement (Supplemental Figure 4), suggesting a specific association between CD57^+^ Tem cells and ILD.

### CD57^+^ Tem cells are cytotoxic effector CD8^+^ T cells distinct from Temra cells.

**Figure 2:**
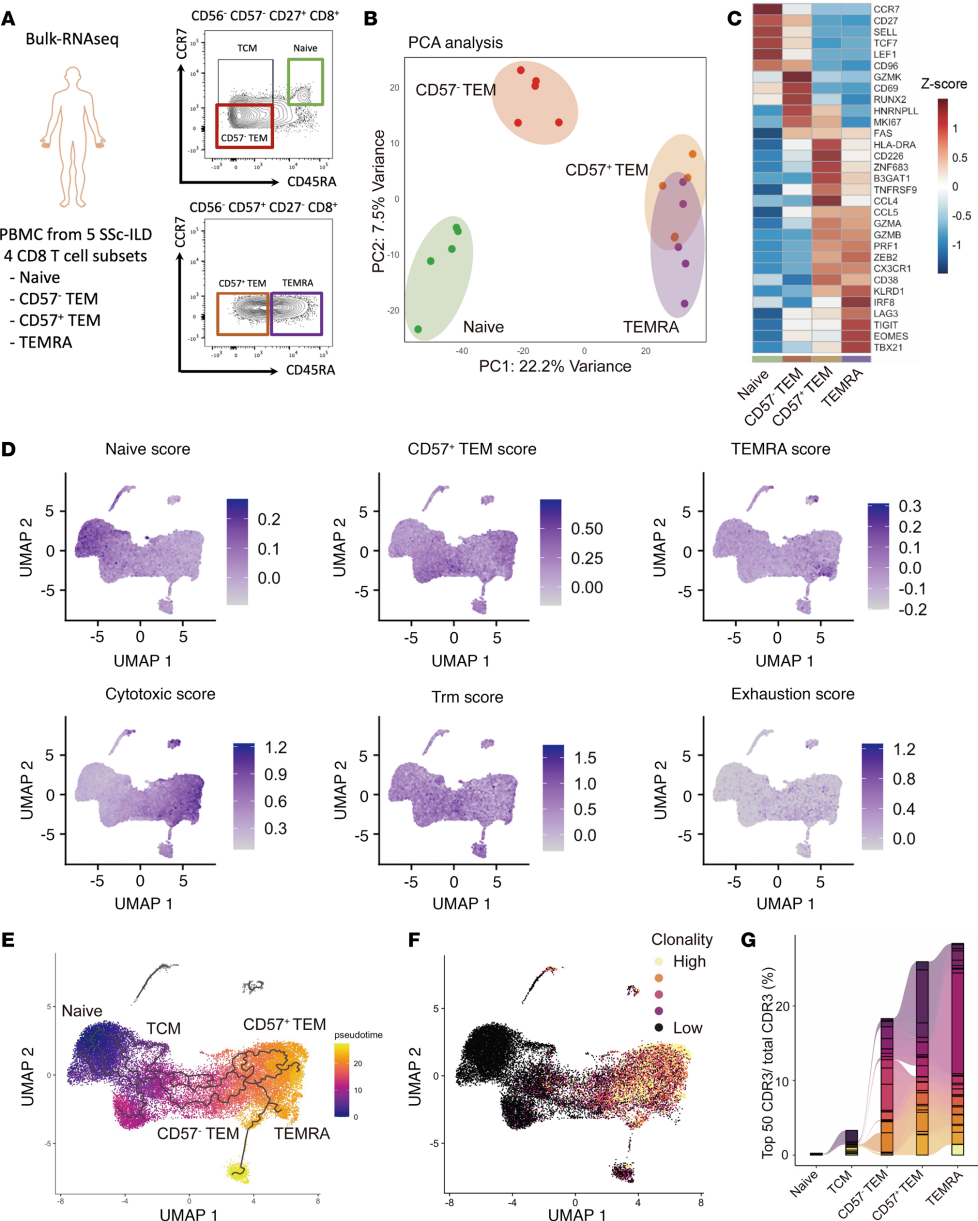
Developmental and clonal relationship between CD57^+^ Tem cells and Temra CD8^+^ T cells. (**A**)Bulk RNA-seq sorting strategy for CD8^+^ T cell populations in this study: naive CD8^+^ T cells (CD57^–^CD27^+^CCR7^+^CD45RA^+^CD56^–^), CD57^–^ Tem cells (CD57^–^CD27^+^CCR7^–^CD45RA^–^CD56^–^), CD57^+^ Tem cells (CD57^+^CD27^–^CCR7^–^CD45RA^–^CD56^–^), and Temra CD8^+^ T cells (CD57^+^CD27^–^CCR7^–^CD45RA^+^CD56^–^) from 5 SSc-ILD donors were sorted. (**B**) PCA plots of naive CD8^+^ T cells, CD57^–^ Tem cells, CD57^+^ Tem cells, and Temra CD8^+^ T cells. (**C**) Heatmap of the expression of selected genes in bulk RNA-seq data from T cell subsets. (**D**) Gene module scores in CD8^+^ T cells in the scRNA-seq data. (**E**) Pseudotime analysis of CD8^+^ T cells in the scRNA-seq dataset. (**F**) Expanded TCR clones depicted onto the transcriptionally defined UMAP visualization. (**G**) Proportions of top 50 clonotypes of total clonotypes in naive, Tcm, CD57^–^ Tem, CD57^+^ Tem, and Temra CD8^+^ T cells.

The above results indicated that CD57^+^ Tem cells were uniquely associated with SSc-ILD prevalence and severity in a pattern not shared with Temra cells. To further assess the transcriptomic signatures of CD57^+^ Tem cells and their differences from Temra cells, we performed bulk RNA-seq of naive, CD57^–^ Tem, CD57^+^ Tem, and Temra CD8^+^ T cells isolated from 5 patients with SSc-ILD (Figure 2A). Principal component analysis (PCA) analysis revealed that CD57^+^ Tem and Temra CD8^+^ T cells were distinct from naive and CD57^–^ Tem cells (Figure 2B). Naive CD8^+^ T cells showed high expression of *CCR7*, *CD27*, *SELL*, *TCF7*, and *LEF1*. CD57^–^ Tem cells had high expression of the early activation marker *CD69* and retained stem memory markers *TCF7* and *LEF1*, alongside evidence of proliferation indicated by MKI67 expression. CD57^–^ Tem cells also displayed high expression of *GZMK*, aligning with our findings from flow cytometric and scRNA-seq analyses (Supplemental Figure 2 and Supplemental Figure 3A). CD57^+^ Tem cells showed elevated expression of cytotoxic molecules and associated features, including *GZMA*, *GZMB*, *PRF1*, *ZEB2*, and *CX3CR1*, along with *TGFB1* (Figure 2C and Supplemental Figure 5). CD57^+^ Tem cells also had high expression of *HLADRA* and *TNFRSF9* (encoding 4-1BB). Temra CD8^+^ T cells also expressed high levels of cytotoxic molecules but uniquely demonstrated elevated expression of *IRF8*, *LAG3*, *TIGIT*, and *EOMES*. Other potentially profibrotic cytokines, including *IL4*, *IL13* (18) and *TGFB2*, were expressed at low levels across all 4 cell populations (Supplemental Figure 5A). Transcriptomics analysis also revealed robust expression of *IFNG* in CD57^+^ Tem and Temra cells, whereas *IL17A* expression was not detected. Consistent with this result, upon anti-CD3 antibody stimulation, CD57^+^ Tem and Temra cells produced IFN-γ, but showed minimal IL-17 expression (Supplemental Figure 5, B and C).

Next, we assessed the expression of these bulk RNA-seq–derived signatures in the scRNA-seq profiling data from patients with SSc to extend the characterization of the cell populations associated with ILD. We established gene module scores for CD57^+^ Tem cells (*n* = 44 upregulated genes, FDR <0.1, Supplemental Table 1) and for Temra cells (*n* = 71 upregulated genes, FDR <0.1, Supplemental Table 2) from the bulk RNA-seq data (Figure 2D). In the scRNA-seq data, CD57^+^ Tem and Temra clusters demonstrated high levels of the respective gene module scores, confirming the distinct transcriptomic signatures of these cells across 2 transcriptional datasets. Cytotoxic gene signatures were highly enriched in the CD57^+^ Tem and Temra cell clusters, with no significant enrichment observed for tissue-resident memory CD8^+^ T cells (Trm) or exhausted T cells. To investigate the developmental relationship between these subsets, we performed pseudotime analysis using Monocle 3 (19, 20). The analysis revealed a differentiation trajectory beginning with naive CD8^+^ T cells, transitioning through T central memory cells (Tcm) and CD57^–^ Tem cells and branching into both CD57^+^ Tem and Temra CD8^+^ T cells (Figure 2E). T cell receptor (TCR) clonality analyses using TCR data captured in the scRNA-seq analysis revealed that both CD57^+^ Tem cells and Temra cells were highly clonally expanded in patients with SSc (Figure 2F), and clonal overlap analysis showed that CD57^–^ Tem, CD57^+^ Tem, Temra, and proliferating cell clusters shared TCR repertoires (Supplemental Figure 3B), with identical TCRs by CDR3 sequence shared among CD57^–^ Tem, CD57^+^ Tem, and Temra CD8^+^ T cells (Figure 2G).

To determine whether the observed clonal expansions were driven by virally reactive T cells, we assessed TCR specificities in all expanded CD8^+^ T cells for reactivity to known viral TCRs (CMV, EBV, influenza). Viral reactivity was evaluated using 2 approaches: (a) exact sequence matching between patient and viral TCRs and (b) grouping of lymphocyte interactions by paratope hotspots (GLIPH2) (21) to identify patient TCRs that significantly shared motifs with viral TCRs. Across the expanded CD8^+^ T cell population, exact matches were rare, and GLIPH2 analysis indicated that most TCRs had no predicted viral reactivity (Supplemental Figure 3C). Comparison of predicted viral TCR reactivity in expanded clones between SSc-ILD and SSc without ILD showed that the vast majority of cells had no viral match in either disease state (Supplemental Figure 3D). This indicates that most clonal expansions likely arise from antigen specificities other than common viral epitopes and may recognize self-antigens associated with SSc or SSc-ILD. Altogether, these findings indicates that cells within CD57^+^ Tem and Temra cell subsets can recognize a common antigen and share a developmental path, either through plasticity across the phenotypes or through a common progenitor state as CD57^–^ cells.

### CD226 augments the cytotoxic function of CD57^+^ Tem cells.

**Figure 3:**
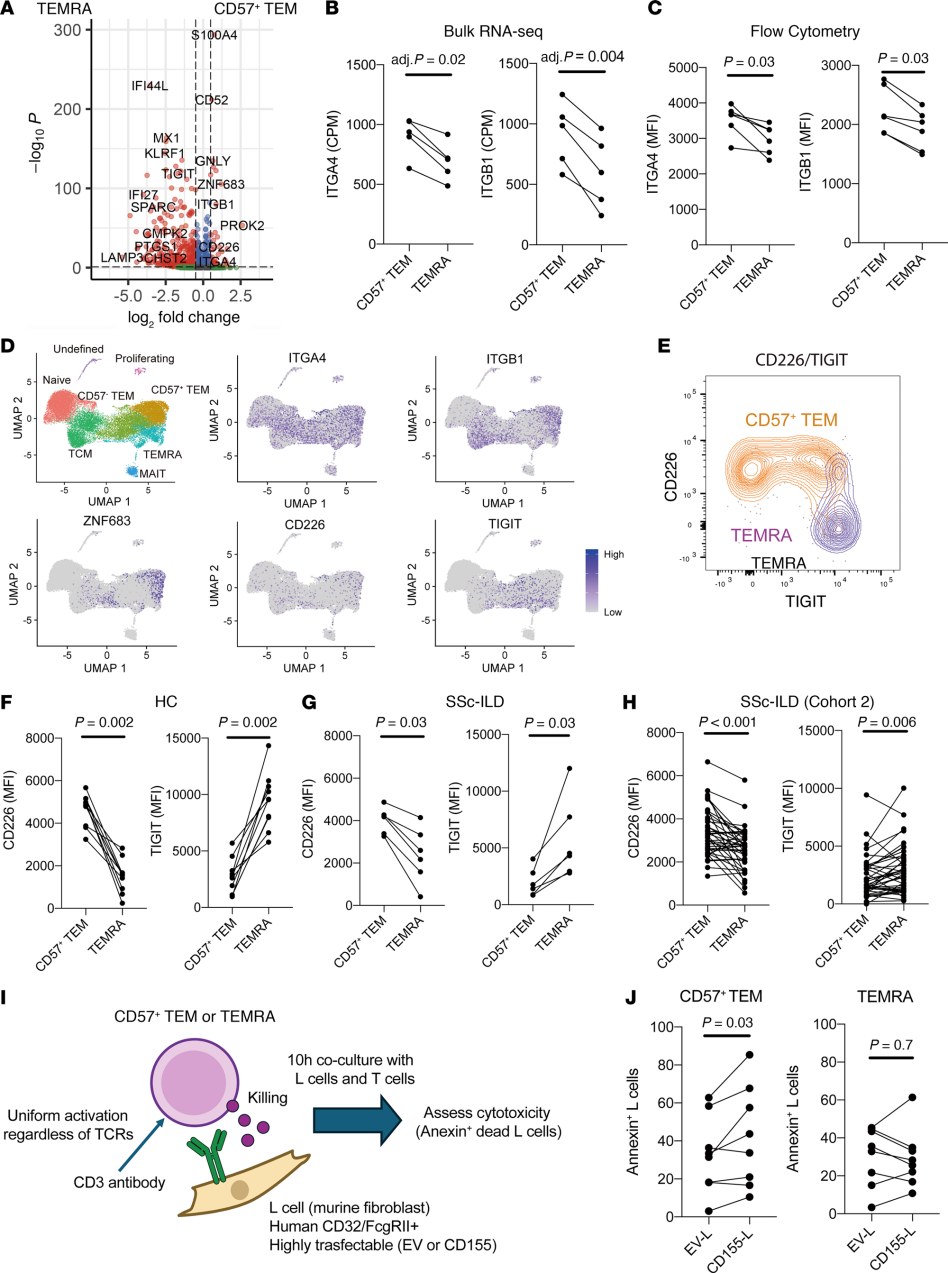
CD226 enhances the activation of CD57^+^ Tem cells. (**A**) DEG analysis between the CD57^+^ Tem cell cluster and the Temra CD8^+^ T cell cluster from the scRNA-seq data. (**B** and **C**) Expression of *ITGA4* and *ITGB1* in CD57^+^ Tem and Temra CD8^+^ T cells from patients with SSc-ILD assessed by bulk RNA-seq (*n* = 5) and flow cytometry (*n* = 6). Wilcoxon test shown. (**D**) Expression of *ITGA4*, *ITGB1*, *ZNF683*, *CD226*, and *TIGIT* in CD8^+^ T cell clusters. (**E**) Representative expression of CD226 and TIGIT on CD57^+^ Tem cells and Temra CD8^+^ T cells by flow cytometry. (**F** and **G**) CD226 and TIGIT expression on CD57^+^ Tem cells and Temra CD8^+^ T cells by flow cytometry. The samples from 11 HCs (**F**) and 6 patients with SSc-ILD (**G**) were used. *P* values in **F** and **G** were determined by Wilcoxon test. (**H**) Validation of CD226 and TIGIT expression in cohort 2 (SSc-ILD, *n* = 41). *P* values were determined by Wilcoxon test. (**I**) Protocol scheme of the cytotoxicity assay to assess the effect of CD155 on CD57^+^ Tem and Temra CD8^+^ T cells. (**J**) Proportion of annexin^+^ L cells with or without CD155 after coculturing with CD8^+^ T cell subsets. CD57^+^ Tem cells and Temra CD8^+^ T cells were collected from 8 HC donors. *P* values were determined by Wilcoxon test.

To identify potential functional differences between CD57^+^ Tem and Temra CD8^+^ T cells, we interrogated differentially expressed genes (DEGs) between these 2 cell clusters in the scRNA-seq data (1,048 DEGs with an FDR <0.1, Figure 3 and Supplemental Tables 3 and 4) and focused on genes encoding surface proteins and known regulators of T cell function. CD57^+^ Tem cells expressed elevated levels of costimulatory molecules such as CD2, CD5, and CD6, suggesting that they may be efficiently activated upon antigen encounter. CD57^+^ Tem cells also had high expression of *ITGA4*, *ITGB1*, *CD226*, and *ZNF683* (encoding HOBIT), while Temra CD8^+^ T cells showed elevated expression of *MX1*, *IFI44*, *IRF8*, and *TIGIT* (Figure 3A). Bulk RNA-seq and flow cytometric analyses confirmed that ITGA4 and ITGB1 (the 2 subunits of VLA4 integrin) were highly expressed in CD57^+^ Tem cells compared with Temra CD8^+^ T cells (Figure 3, B and C). In addition, CD226 and TIGIT showed distinct patterns of expression between CD57^+^ Tem and Temra cells (Figure 3, D and E). Notably, the costimulatory receptor CD226 and the inhibitory receptor TIGIT shared the same ligands, CD155 and CD112. Flow cytometric analysis verified that CD226 expression was higher in CD57^+^ Tem cells, whereas TIGIT expression was higher in Temra CD8^+^ T cells, in both patients with SSc-ILD and controls (Figure 3, F and G). CD57^+^ Tem cells from patients with SSc-ILD in cohort 2 also showed higher expression of CD226 and lower expression of TIGIT compared with Temra cells (Figure 3H).

CD226 and TIGIT share the same ligands, CD155 and CD112, but can transmit opposing signals: CD226 provides an activating signal, whereas TIGIT sends an inhibitory signal (21, 22). Based on this, we hypothesized that the presence of CD155/CD112 may preferentially activate CD57^+^ Tem cells over Temra CD8^+^ T cells. To evaluate the potential functional effects of CD155 on CD57^+^ Tem and Temra CD8^+^ T cells, we applied a previously established cytotoxicity assay (23). CD57^+^ Tem or Temra CD8^+^ T cells were cocultured with murine fibroblast L cells loaded with an anti-CD3 antibody to activate CD8^+^ T cells and measure their killing activity (Figure 3I). To assess the specific effect of CD155, we compared the cytotoxicity induced by target L cells transduced to express human CD155 versus control transduced target L cells. CD57^+^ Tem cells demonstrated significantly increased cytotoxic activity when cocultured with CD155^+^ L cells, whereas the cytotoxicity of Temra CD8^+^ T cells was not altered (Figure 3J). These results suggest that CD57^+^ Tem cells differ from Temra cells in their regulation by CD155, with CD155 providing a stimulating effect on the cytotoxic function in the CD57^+^ Tem cell population expanded in patients with SSc-ILD.

### CD57^+^ Tem cells are infiltrated and expanded in patients with SSc-ILD.

**Figure 4:**
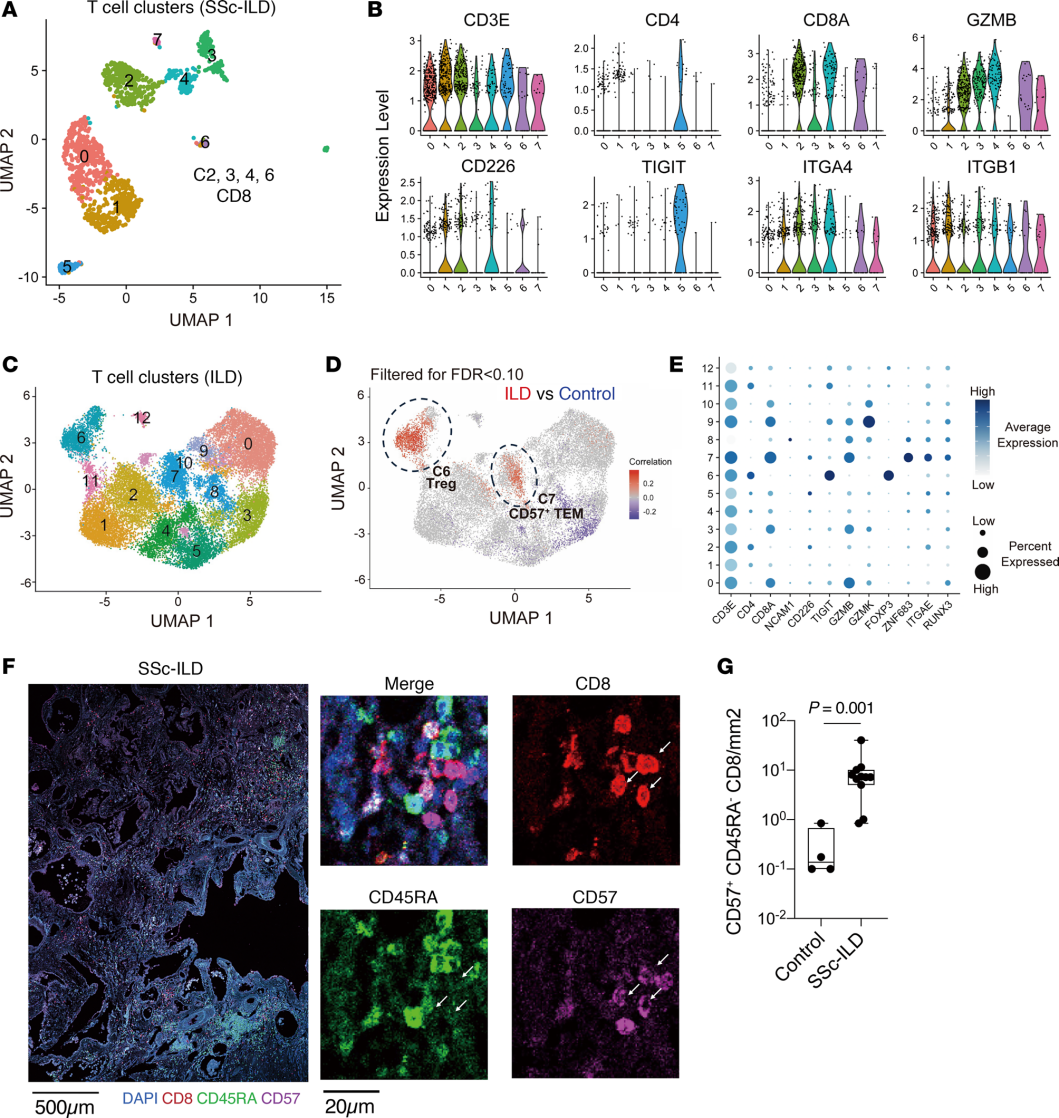
CD57^+^ Tem cells are expanded in SSc-ILD lung tissues. (**A**) UMAP clustering of T cells in lung tissue scRNA-seq data from 4 SSc-ILD donors (24). (**B**) Gene expression of the T cell clusters from **A**. (**C**) UMAP clustering of T cells in lung tissue scRNA-seq data generated from 66 patients with ILD and 48 control donors (25). (**D**) CNA of CD8^+^ T cells from the scRNA-seq data, adjusting for age and sex. Red indicates cell neighborhoods enriched in ILD samples. (**E**) Gene expression of the CD8^+^ clusters in the scRNA-seq data in **C**. (**F**) Representative images of CD57^+^, CD8^+^, and CD45RA^+^ staining of SSc-ILD lung tissue. Scale bars: 500 μm and 20 μm. (**G**) Quantification of CD57^+^CD45RA^–^CD8^+^ in tissue stainings. (HCs: *n* = 4, SSc-ILD: *n* = 11). *P* value was determined by Mann-Whitney *U* test. Box plots represent median and interquartile range (25th-75th percentiles), with whiskers indicating minimum and maximum values.

We next sought to determine whether CD57^+^ Tem cells are present in the lungs of patients with SSc-ILD. We first analyzed published scRNA-seq data from lungs of 4 patients with SSc-ILD (24), which revealed the presence of CD8^+^ T cells expressing *GZMB* and *CD226* (Figure 4, A and B), consistent with the phenotype expanded in the circulation of patients with SSc-ILD. To assess the accumulation of CD8^+^ T cells across a larger range of ILD samples, we analyzed a published lung tissue scRNA-seq dataset generated from 114 donors (*n* = 66 ILD samples and *n* = 48 control samples) (25). In this dataset, 3 of 66 ILD samples were derived from patients with connective tissue disease–associated ILD, whereas the remainder included other forms of ILD. CNA of T cell clusters indicated that C6 (Tregs) and C7 (cytotoxic CD8^+^ T cells resembling CD57^+^ Tem cells) were highly enriched in ILD lung samples compared with controls (Figure 4, C and D). Interestingly, unlike CD57^+^ Tem cells in blood, C7 (CD57^+^ Tem) exhibited high expression of *ITGAE* (CD103) and *RUNX3* (Figure 4E), markers of Trm cells (26–29), suggesting that CD57^+^ Tem cells may acquire Trm features in the lung, allowing them to maintain residence in the tissue. To confirm the presence of CD57^+^CD8^+^ T cells in SSc-ILD lungs unambiguously at the protein level, we quantified CD57^+^ Tem cells by immunofluorescence microscopy in SSc-ILD and control lung samples collected via video-assisted thoracoscopic surgery (VATS) or as lung explants. We found that CD57^+^ Tem cells were abundant in lung tissue from patients with SSc-ILD compared with tissue from controls (Figure 4, F and G). Together, these results suggest that the CD8^+^ T cells infiltrating SSc-ILD lung tissues share similar characteristics with the expanded population of CD57^+^ Tem cells in the circulation.

### Expansion of CD155^+^VCAM1^+^ endothelial cells in multiple ILD cohorts.

**Figure 5:**
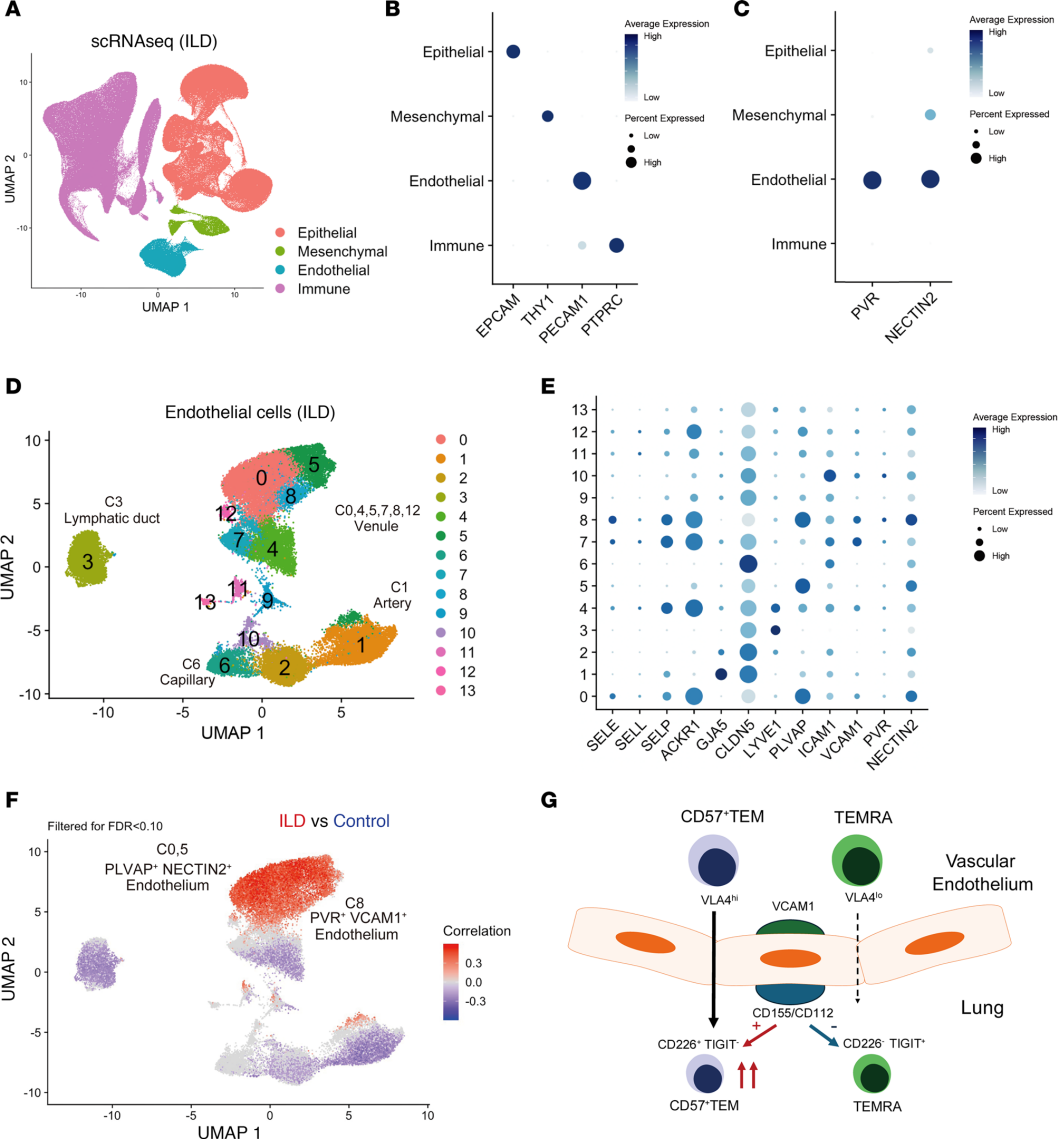
Endothelial cells are the main source of CD155 and CD112 in ILD tissues. (**A**) UMAP clustering of all cells in lung tissue scRNA-seq data generated from 66 patients with ILD and 48 control donors (25). (**B**) Expression of EPCAM, THY1, PECAM1, and PTPRC in the cell subsets from **B**. (**C**) Expression of CD155/PVR and CD112/NECTIN2 in cell subsets from **B**. (**D**) UMAP clustering of endothelial cells in scRNA-seq data from **B**. (**E**) Expression of CD155/PVR and CD112/NECTIN2 in endothelial clusters. (**F**) CNA of endothelial cells from **B**, comparing ILD versus control samples. Red indicates cell neighborhoods that were enriched in the ILD samples. (**G**) Proposed model of CD57^+^ Tem cell–driven pathogenic mechanisms in SSc-ILD.

Given the evidence that ligands for CD226 augment the activation of CD57^+^ Tem cells, we assessed the expression of the ligands CD155 and CD112 in SSc-ILD lung tissue. *CD155* (also known as poliovirus receptor [*PVR*]) was detected in lymphatic vessels and vascular endothelial cells in the lungs of patients with SSc-ILD (Supplemental Figure 6) (24). *CD112* (also known as nectin cell adhesion molecule 2 [*NECTIN2*]) was not detected in this dataset. In the larger dataset of cells across different ILD lung samples, both *CD155/PVR* and *CD112/NECTIN2* were expressed highly in endothelial cells (Figure 5, A–C) (25). CNA of the broader ILD dataset focused on the endothelial cells revealed that 3 venule clusters C0, C5, and C8 were highly enriched in the ILD samples (Figure 5, D and E). Notably, these 3 clusters highly expressed *NECTIN2* and plasmalemma vesicle–associated protein (*PLVAP*). *PLVAP* forms a diaphragm that regulates vascular permeability (30–32). Interestingly, C8 also showed high expression of *CD155/PVR* and *VCAM1* (Figure 5F), the latter of which is a receptor for the integrin VLA4 (33). A similar endothelial cell cluster highly expressing *VCAM1* and *CD155/PVR* was also detected in the Idiopathic Pulmonary Fibrosis (IPF) Cell Atlas (Supplemental Figure 7) (34, 35). Given that CD57^+^ Tem cells highly express VLA4, the interaction between VLA4 on CD57^+^ Tem and VCAM1 on venular endothelial cells may promote the migration of CD57^+^ Tem cells into lung tissue. This migration, followed by CD155-mediated activation, may enhance the cytotoxic activity of CD57^+^ Tem (Figure 5G).

## Discussion

In this study, we used extensive mass cytometry and scRNA-seq profiling data from a well-characterized cohort of patients with SSc to identify a specific cytotoxic CD8^+^ T cell subset that is expanded in the circulation of patients with SSc-ILD and accumulates within SSc-ILD lungs. The expanded CD57^+^ Tem population was distinguished from Temra CD8^+^ T cells by a distinct transcriptomic signature and high expression of VLA4 integrin and CD226, which may allow activation by and targeting of endothelial cells. Recognition of this expanded CD57^+^ Tem cell population with pathologic function provides a specific feature of immune activation in SSc-ILD that may serve as a therapeutic target and a candidate biomarker for SSc-ILD.

Here, we demonstrate that CD57^+^CD8^+^ Tem cells were expanded in multiple cohorts of patients with SSc-ILD. These findings extend prior observations that CD57^hi^CD4^+^ CTLs are increased in patients with SSc, and among these patients, those with a higher proportion of CD57^hi^CD4^+^ CTLs have a higher prevalence of lung disease complications (12, 13). CD57^+^ Tem cells highly expressed T cell activation molecules such as CD2, CD5, and CD6. These molecules are known to participate in costimulatory signaling and T cell–APC interactions, suggesting that CD57^+^ Tem cells may have a transcriptional profile of enhanced activation potential. Endothelial cells may be a key driver of the expansion of this immune cell subset. Our observation of high expression of VLA4 and CD226 on CD57^+^ Tem cells provides evidence for a potential interaction between cytotoxic T cells and venular endothelial cells, which express both VCAM1 and CD155, as a target of the cytotoxic T cell response. Considering that blood flow velocity is high in arterial regions, where it is difficult for immune cells to adhere to the endothelium, it is reasonable to propose that endothelial cells in the post-capillary area may support the infiltration of CD57^+^ Tem cells. Escalante et al. reported that CD155 expression on human umbilical vein endothelial cells (HUVECs) is enhanced by IFN-γ and IL-1 (36). This mechanism may act as an immune checkpoint for T cells, such as Temra cells, expressing TIGIT. In contrast, CD57^+^ Tem cells with low expression of TIGIT but high CD226 expression may evade CD155-dependent immunotolerance and instead exert proinflammatory effects via CD226 signaling. This activating effect of endothelial cells may enhance the cytotoxic injury mediated by CD57^+^ Tem cells.

One of the distinguishing features of CD57^+^ Tem cells is the high expression of *ZNF683*. *ZNF683*, also known as Hobit, is a key transcription factor for Trm cells, which reside in tissues and respond rapidly to viral and bacterial infections in the lungs (37–39). Interestingly, CD57^+^ Tem cells in lung tissue showed high expression of ITGAE (also known as CD103) and RUNX3, which are characteristic of Trm cells (26–29). In contrast, CD57^+^ Tem cells in peripheral blood of patients with SSc-ILD did not express these markers. We hypothesize that circulating CD57^+^ Tem cells may acquire Trm-like characteristics upon infiltration into the lungs, allowing them to reside and manifest their effector cytotoxic function in affected tissues, thus contributing to pathogenetic injury and chronic inflammation in ILD. Furthermore, ZNF683-expressing CD8^+^ T cells in the blood were reported to be enriched for antigen reactivity in a case of kidney allograft rejection following immune checkpoint inhibitor therapy (40). The TCRs from alloreactive CD8^+^ T cells infiltrating the rejected kidneys showed extensive overlap with the TCRs of ZNF683-expressing CD8^+^ T cells in blood. These findings further support the concept that circulating ZNF683-expressing CD8^+^ T cells migrate to sites of inflammation, where they contribute to tissue damage.

The detection of expanded CD57^+^ Tem cells in the circulation of patients with SSc-ILD nominates this cellular feature as a potential biomarker to identify patients at risk for progressive ILD or to track the extent of the ongoing pathologic immune activation. A key limitation of our study is its cross-sectional design. Although we observed an association between CD57^+^ Tem cell frequency and ILD severity, longitudinal studies will be required to determine whether this cell population serves as a reliable biomarker for disease progression or treatment response. We believe our findings are therefore hypothesis generating and warrant prospective validation in the future. While circulating CD57^+^ Tem cells exhibited high expression of cytotoxic molecules, and analysis of publicly available datasets confirmed the presence of similar cell populations in the lungs, to establish a more direct link between circulating and lung-resident cell populations, studies incorporating paired blood and lung samples will also be necessary. Furthermore, cytotoxicity assays were performed using murine fibroblast cell lines to express human CD155, which may not fully recapitulate the physiological interactions between human T cells and lung-resident target cells. While this system allowed us to assess the role of CD155/CD226 signaling, future studies using primary human lung endothelial cells, organoids, or in vivo animal studies will be important to validate these findings in a more physiologically relevant setting. Although our study focused on the association between CD57^+^ T cells and ILD, we did not investigate whether the presence or severity of other key disease manifestations, particularly gastroesophageal reflux disease, may contribute to expansion and activation of this T cell subset in the lungs of subjects with SSc-ILD. Nonetheless, the study highlights a specific, clonally expanded CD8^+^ T cell population with plausible pathologic function that is associated with SSc-ILD. As the presence of ILD carries an important prognostic value not only in SSc but also in other autoimmune rheumatic conditions such as rheumatoid arthritis and myositis, it will be of interest to determine whether circulating CD57^+^ Tem cells are similarly increased in patients with ILD affected by these conditions and whether this cellular subset can be targeted for therapeutic benefit.

## Methods

### Sex as a biological variable.

We accounted for sex as a biological variable by adjusting for sex as a confounding factor, ensuring it was appropriately considered in our analyses. We compared the frequencies of CD57^+^ Tem cells between male and female patients and confirmed that there were no significant differences (Supplemental Figure 2D).

### Sample collection.

PBMCs were collected from 67 patients with SSc at UCSF and from 15 patients with SSc and 18 HCs at BWH. All patients with SSc fulfilled the 2013 American College of Rheumatology/EULAR criteria for SSc (41). The presence of ILD was confirmed by radiographic evidence of pulmonary fibrosis on HRCT conducted in patients exhibiting a FVC of less than 80%, an absolute decline of the FVC (L) of greater than 10% over 2 consecutive assessments, or evidence of bibasilar crackles at lung auscultation. Pulmonary arterial hypertension (PAH) was confirmed with right heart catheterization by mean pulmonary artery pressure (mPAP) above 25 mmHg, peripheral vascular resistance of greater than 3 Wood units, and pulmonary capillary wedge pressure below 15 mmHg, as the updated 2022 hemodynamic definition of PAH was not yet established at the time of patient enrollment (42). HCs were screened for any evidence of autoimmune systemic disease, neoplasm, or lung pathology. Blood samples were collected in heparin tubes, and PBMCs were isolated by density centrifugation using Ficoll-Hypaque (Cytiva) in 50 mL conical tubes. PBMCs were cryopreserved in 10% DMSO-containing solution.

### Mass cytometry.

Cryopreserved PBMCs were thawed into complete culture media (RPMI 1640 Medium supplemented with 5% heat-inactivated FBS, 1 mM GlutaMAX, 10 mM HEPES, and penicillin-streptomycin). Cells were counted, and 0.2 × 10^6^ to 2 × 10^6^ cells from each sample were transferred to a 96-well conical bottom polypropylene plate for staining. After the cells were transferred to the plate, the viability staining reagent cisplatin at a dilution of 1:1,000 was added to the cells directly for 2 minutes and then diluted with culture media. After centrifugation, a human Fc receptor–blocking agent was added at a 1:100 dilution with cell staining buffer for 10 minutes, followed by incubation with metal-conjugated antibodies for 30 minutes at room temperature. Antibodies were obtained from the Harvard Medical Area cytometry by time-of-flight mass spectrometry (CyTOF) Antibody Resource and Core and Fluidigm. Cells were then fixed in 4% paraformaldehyde for 10 minutes before permeabilization with the FoxP3/Transcription Factor Staining Buffer Kit (eBioscience). Cells were incubated in Transcription Factor Fix/Perm Buffer for 30 minutes before barcoding. Cells were then barcoded using the Cell-ID 20-Plex Pd Barcoding kit (Fluidigm) and pooled together into 1 tube. The metal-conjugated intracellular antibody mix was then added into the tube, and cells were incubated for 30 minutes. Cells were then fixed with 4% paraformaldehyde for 10 minutes and then washed out and resuspended in a cell-staining buffer and left at 4°C overnight. The next day, DNA was labeled for 20 minutes with iridium intercalator solution (Fluidigm). Samples were washed and then counted in the presence of EQ Four Element Calibration beads at a final concentration of 1 × 10^6^/mL. Samples were acquired on a Helios CyTOF Mass Cytometer. Mass cytometric data were generated for PBMC samples from patients with SSc and HCs using a 39-marker mass cytometry panel designed to identify T cell subsets (Supplemental Table 6). Samples were processed in 5 batches, with 20 barcoded samples in each batch. Patient samples were analyzed in barcoded batches, with each batch randomized to include samples from both HCs and patients with SSc with different degrees of lung involvement. The raw FCS files were normalized together using bead standard normalization to minimize batch effects and were debarcoded for analysis. Live cells were gated as DNA^+^ Live^+^ Beads^–^ using FlowJo (BD), and downstream analysis was performed using the Seurat R package (version 5.1.0) (43). After calculating principal components (PCs), batch correction was applied using the Harmony R package (44). Uniform manifold approximation and projection (UMAP) visualization and clustering were then performed. Cells associated with SSc-ILD were identified using CNA, adjusting for age and sex (17).

### scRNA-seq.

Sixty-seven cryopreserved PBMC samples from the UCSF scleroderma cohort were run in 2 batches processed using Chromium Next GEM Single Cell 3 Kit (10X Genomics) and 4 batches processed using Chromium Next GEM Single Cell 5 Kit (10X Genomics) for data generation. Standard 10X protocols were used for the library preparation. The FASTQ files were aligned to the GRCh38 human reference genome using Cell Ranger (10X Genomics). Demultiplexing was performed using freemuxlet (https://github.com/statgen/popscle/), an extension of demuxlet (45) with genotype information. Doublets and cells whose donor origins were not identified were excluded. The files were processed with the Seurat R package (version 5.1.0). For the quality control (QC) filter, we removed cells that expressed fewer than 400 genes, more than 4,000 genes, or contained more than 15% of total unique molecular identifiers (UMIs) associated with mitochondrial genes. After QC, mRNA expression was log normalized (scale.factor = 10,000). Then, the highly variable genes, selected through variance-stabilizing transformation, were scaled. Next, PCs were calculated on the basis of mRNA data, and samples were batch corrected using the Harmony R package (44). UMAP visualization and clustering were performed, and CD8^+^ T cell clusters were extracted. Consequently, a total of 351,481 analyzable cells, including 29,162 CD8^+^ T cells, from 58 donors were detected (Supplemental Table 5). Sequencing reads for the TCR library were processed through the Cell Ranger workflow. Reads were aligned to a TCR reference (vdj-GRCh38), productive contigs were filtered, and CDR3 sequences were identified for each cell. Cells with matching CDR3alpha and CDR3beta were grouped into the same clonotype. Each cell’s TCR was then paired with its transcriptomic data by matching cell barcodes. These files were then analyzed using the scRepertoire package (46).

### Viral reactivity analysis.

Expanded CD8^+^ T cells (clone size >1) were identified from the single-cell TCR dataset after excluding naive cells, leaving 10,304 cells for analysis. Reference virus-reactive TCRs were obtained from the McPAS (47) and VDJdb (48) databases and filtered to retain only sequences annotated for EBV, CMV, or influenza. Analyses were restricted to CDR3β amino acid sequences. Exact matches between patient and reference TCRs were identified by comparing CDR3β sequences directly. Predicted viral reactivity was evaluated using GLIPH2 (49), which was run with patient TCRs, viral reference TCRs, and HLA genotypes inferred from single-cell FASTQ files using the arcasHLA package. GLIPH2 output was filtered to retain groups with a Fisher score below 0.05.

### Flow cytometric analysis.

After cryopreserved PBMCs were thawed into complete culture media, they were washed with PBS and stained with violet viability dye (Thermo Fisher Scientific) on ice for 15 minutes. After washing with PBS, cells were stained with flow antibodies on ice for 30 minutes. The flow antibodies used in this study are listed in Supplemental Table 7. When performing intracellular staining, cells were fixed with Foxp3 Fixation/Permeabilization (eBioscience) for 30 minutes at room temperature. After washing with Permeabilization Buffer (eBioscience), cells were stained with anti-GZMB and anti-GZMK antibodies on ice for 30 minutes. After washing twice in 1× eBioscience Permeabilization Buffer and passing through a 70 μM filter, data were acquired on a BD Fortessa analyzer using FACSDiva software and analyzed using FlowJo.

### Bulk RNA-seq.

After cryopreserved PBMCs from 5 SSc-ILD donors were thawed into complete culture media, PBMCs were washed with PBS and stained with violet viability dye (Thermo Fisher Scientific) on ice for 15 minutes. After washing with PBS, cells were stained with flow antibodies on ice for 30 minutes. After washing with 1% BSA in PBS, approximately 20,000 naive CD8^+^ T cells (Live^+^CD3^+^CD8^+^CD56^–^CD57^–^CD27^+^CCR7^+^CD45RA^+^), CD57^–^ Tem cells (Live^+^CD3^+^CD8^+^CD56^–^CD57^–^CD27^+^CCR7^–^CD45RA^–^), CD57^+^ Tem cells (Live^+^CD3^+^CD8^+^CD56^–^CD57^+^CD27^–^CCR7^–^CD45RA^–^), and Temra CD8^+^ T cells (Live^+^ CD3^+^CD8^+^CD56^–^CD57^+^CD27^–^CCR7^–^CD45RA^+^) were directly sorted using a 5L BD FACSAria Fusion cell sorter into RLT lysis buffer kept on ice. Cells were sorted through an 85 μM nozzle. After extracting RNA using the RNeasy Kit (Qiagen, 74104), the samples were submitted to Molecular Biology Core Facilities at Dana-Farber Cancer Institute, and data were generated using mRNAseq Smart-Seq version 4 (50). Raw reads were mapped using the STAR method protocol (https://github.com/alexdobin/STAR). Raw read counts were analyzed by R (version 4.2.0) using DESeq2 (51). After normalizing RNA expression, PCA was performed using prcomp and plotted with ggtools2. Analysis of DEGs was carried out using DESeq2, and volcano plots generated using EnhancedVolcano (https://github.com/kevinblighe/EnhancedVolcano).

### Tissue CD57^+^ Tem cell staining.

Unstained formalin-fixed, paraffin-embedded (FFPE) lung tissue samples were obtained from the BWH pathology core and USCF. After deparaffinization with D-limonene (3 minutes twice) and rehydration with 100% (3 minutes), 95% (3 minutes), and 70% (3 minutes twice) ethanol, antigen retrieval was performed using Antigen Retrieval Buffer (ab93678) at 95°C for 10 minutes. After blocking with PBS and 3% BSA for 30 minutes, the cells were stained with CD57 (Invitrogen, Thermo Fisher Scientific, MA5-12008, ×100), CD8^+^ (Invitrogen, Thermo Fisher Scientific, MA5-14548, ×25), CD45RA (BioLegend, 304102, ×100) primary antibodies in 3% BSA plus 1% Triton-X-100 for 1 hour at room temperature. After washing with PBS, the slides were stained with AF488, AF555, and AF647 secondary antibodies for 1 hour at room temperature. After washing with PBS, DAPI Fluoromount-G (Southern Biotech, no. 0100-20) was mounted on the slides, and the samples were covered with coverglasses. Immunofluorescence images were taken with a Zeiss LSM 800 and then analyzed by QuPath (52).

### Intracellular staining.

Plates were coated with SK7 (5 μg/mL in PBS) overnight at 4°C and washed the following day with PBS. Total CD8^+^ T cells were purified from PBMCs using the MojoSort Human CD8^+^ T Cell Isolation Kit (BioLegend) and seeded at 2 **×** 10^5^ cells per well in the coated plates. Monensin (BioLegend) was added at the time of seeding, and cells were stimulated for 5 hours. After the stimulation, cells were incubated with a viability dye (Zombie, BioLegend) for 10 minutes at room temperature, followed by surface marker staining for 15 minutes at 4°C. After fixation for 20 minutes at room temperature, cells were permeabilized and stained with antibodies against IL-17A and IFN-γ for 20 minutes. Samples were then washed, and data were acquired on a BD Fortessa analyzer using FACSDiva software and analyzed using FlowJo.

### Cytotoxicity assays.

CD32-expressing L cells were provided by Megan Levings (University of British Columbia, Vancouver, Canada). CD8^+^ T cells were isolated from collars with a MACS Total CD8^+^ isolation kit (Miltenyi Biotec) and subsequently sorted with a 5-laser BD FACSAria Fusion cell sorter for CD57^+^ Tem and Temra cells. CD32-expressing L cells were incubated with an agonist anti-CD3 antibody (SK7, Thermo Fisher Scientific) for 30 minutes on ice and then plated with sorted CD8^+^ T cells at a ratio of 3:1 CD8^+^ T cells/L cells. After 10 hours, the T cells and L cells were stained with Annexin V APC and 7-AAD in Annexin Binding Buffer (BioLegend) and analyzed on a BD CantoII analyzer.

### Statistics.

A Mann-Whitney *U* test was used for comparisons between 2 groups and a Kruskal-Wallis with Dunn’s multiple-comparison test for comparisons between 3 groups. Wilcoxon test was used for paired samples. Correlation analysis was performed using Spearman’s test. A *P* value of less than 0.05 was considered significant. Box plots represent median and interquartile range (25th-75th percentiles), with whiskers indicating minimum and maximum values.

### Study approval.

The study was approved by the IRB at UCSF (approval no. 15-16463) and BWH (approval no. 2014P002558). All patients were consented for the collection of blood samples and clinical information.

### Data availability.

Values for all data points in graphs are reported in the Supporting Data Values file. Bulk RNA-seq (accession number: phs004443.v1.p1) and scRNA-seq data (accession number: phs004454.v1.p1) generated in this study are deposited in dbGaP.

## Author contributions

TS conceived the project, performed experiments, analyzed data, and wrote the manuscript. YC, JS, RA, and KET analyzed transcriptomics data. KH and KEM assisted with the in vitro experiments. ME and PFD collected clinical information. NB participated in study design and data interpretation. PW, MK, and EYK collected tissue samples for histology. FB and DAR conceived the project, supervised the work, recruited the patients, and wrote the manuscript. All authors discussed the results and revised the manuscript.

## Conflict of interest

DAR reports grant support from Janssen and Bristol Myers Squibb and reports receiving personal fees from AstraZeneca, Pfizer, Merck, Amgen, Scipher Medicine, GlaxoSmithKline, and Bristol Myers Squibb. He is coinventor on a patent using T peripheral helper cells as a biomarker of autoimmune diseases (“Compositions and methods relating to T peripheral helper cells in autoantibody-associated conditions”). PW reports receiving grants and personal fees from Boehringer Ingelheim; honoraria for lectures from Boehringer Ingelheim and Roche; grants and personal fees from Sanofi; and contract/grant support from Pliant Therapeutics.

## Funding support

This work is the result of NIH funding, in whole or in part, and is subject to the NIH Public Access Policy. Through acceptance of this federal funding, the NIH has been given a right to make the work publicly available in PubMed Central.

- Department of Defense Scleroderma Research Program Translational Research Partnership (SL200016P1, to FB and DAR).
- Doris Duke Charitable Foundation Clinical Scientist Development Award.
- Burroughs Wellcome Fund Career Award in Medical Sciences.
- National Institute of Arthritis and Musculoskeletal and Skin Diseases (NIAMS), NIH (P30-AR-070253, to DAR).
- Kao Multidisciplinary Scleroderma Program at Cedars-Sinai (to FB).
- NIH grant (to PW).

## Supplementary Material

Supplemental data

Supporting data values

## Figures and Tables

**Table 1:**
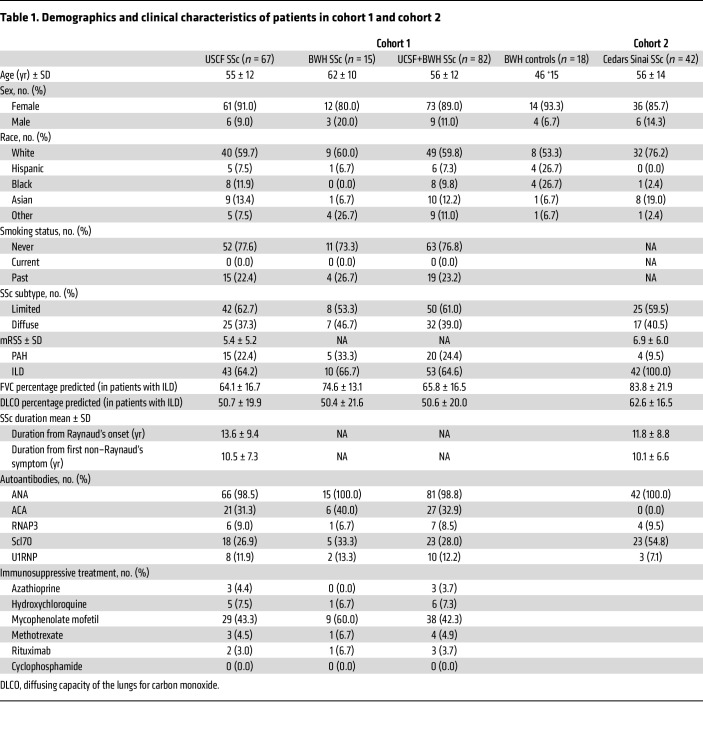
Demographics and clinical characteristics of patients in cohort 1 and cohort 2

## Version 1 02/17/2026

In-Press Preview

## Version 2 05/01/2026

Electronic publication
