# Supplementary material for: A CD57+CD8+ T cell subset links T cell cytotoxicity to fibrotic lung disease in systemic sclerosis
Source: J Clin Invest. 2026 Feb 17;136(9):e194288. doi: 10.1172/JCI194288 (PMC13132381; doi:10.1172/JCI194288)
Supplement: Supplemental data [file jci-136-194288-s057.pdf]

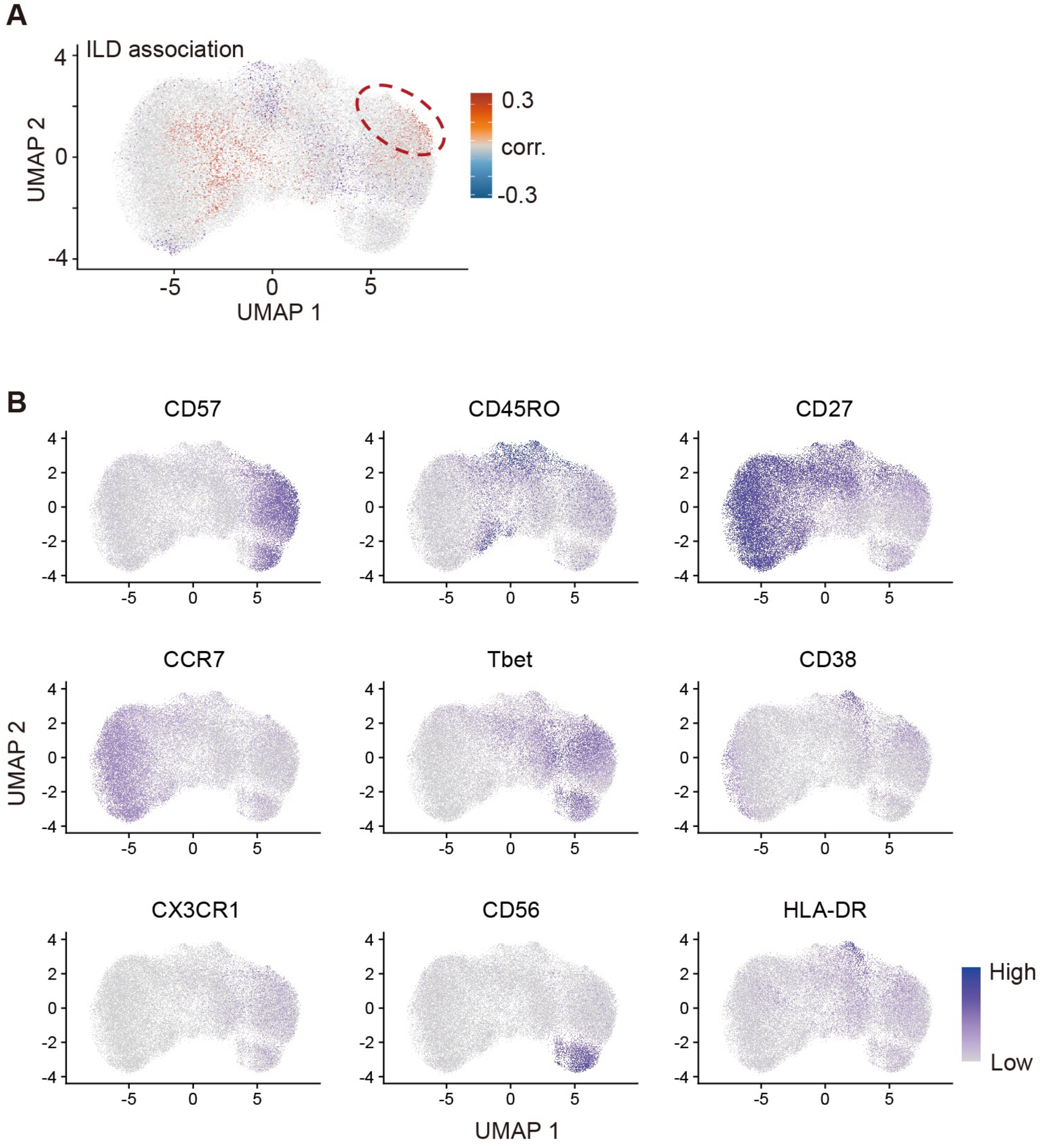


935 **Supplemental Figure 1. Marker expression of CD57$^{+}$ TEM in mass cytometry**

**A.** CNA using CD8 T cells from HC and SSc-ILD patients, adjusting for age and sex. Red indicates cell neighborhoods enriched in SSc-ILD patients. **B.** Marker expression in T cells in mass cytometry data.

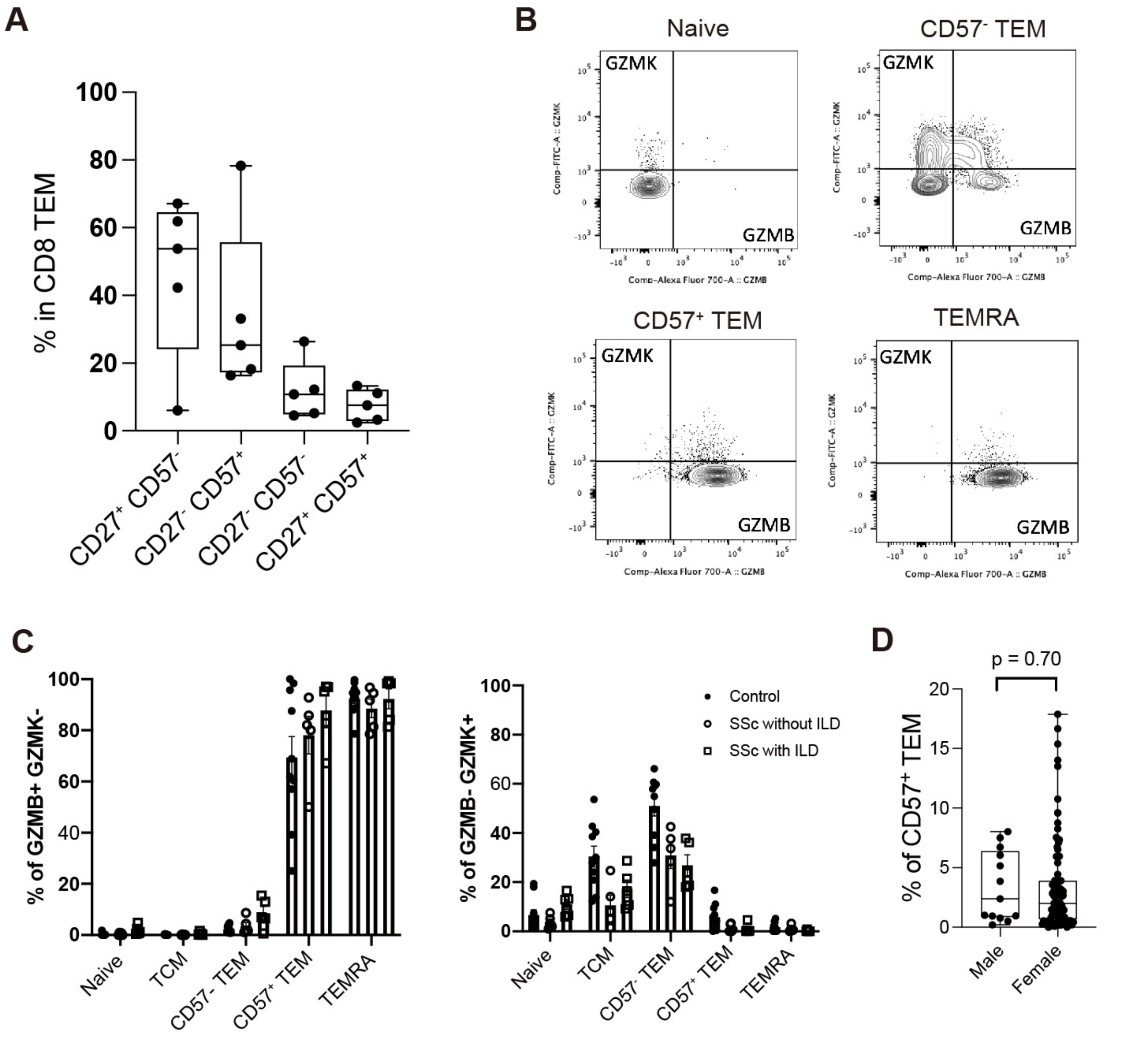


**Supplemental Figure 2. Characteristics of CD57$^+$ TEM.**

**A**. Frequencies of CD27$^+$ CD57$^-$ (CD57$^-$ TEM), CD27$^+$ CD57$^+$, CD27$^-$ CD57$^-$, and CD27$^-$ CD57$^+$ (CD57$^+$ TEM) in CD8 TEM (n = 5, SSc-ILD). **B, C.** Intracellular staining of granzyme B and granzyme K in naive, TCM, CD57$^-$ TEM, CD57$^+$ TEM, and TEMRA CD8 T cells. HC: n = 10, SSc-non ILD: n = 5, SSc-ILD: n = 5. **D.** Comparison of CD57+TEM between males and females. Mann-Whitney U test.

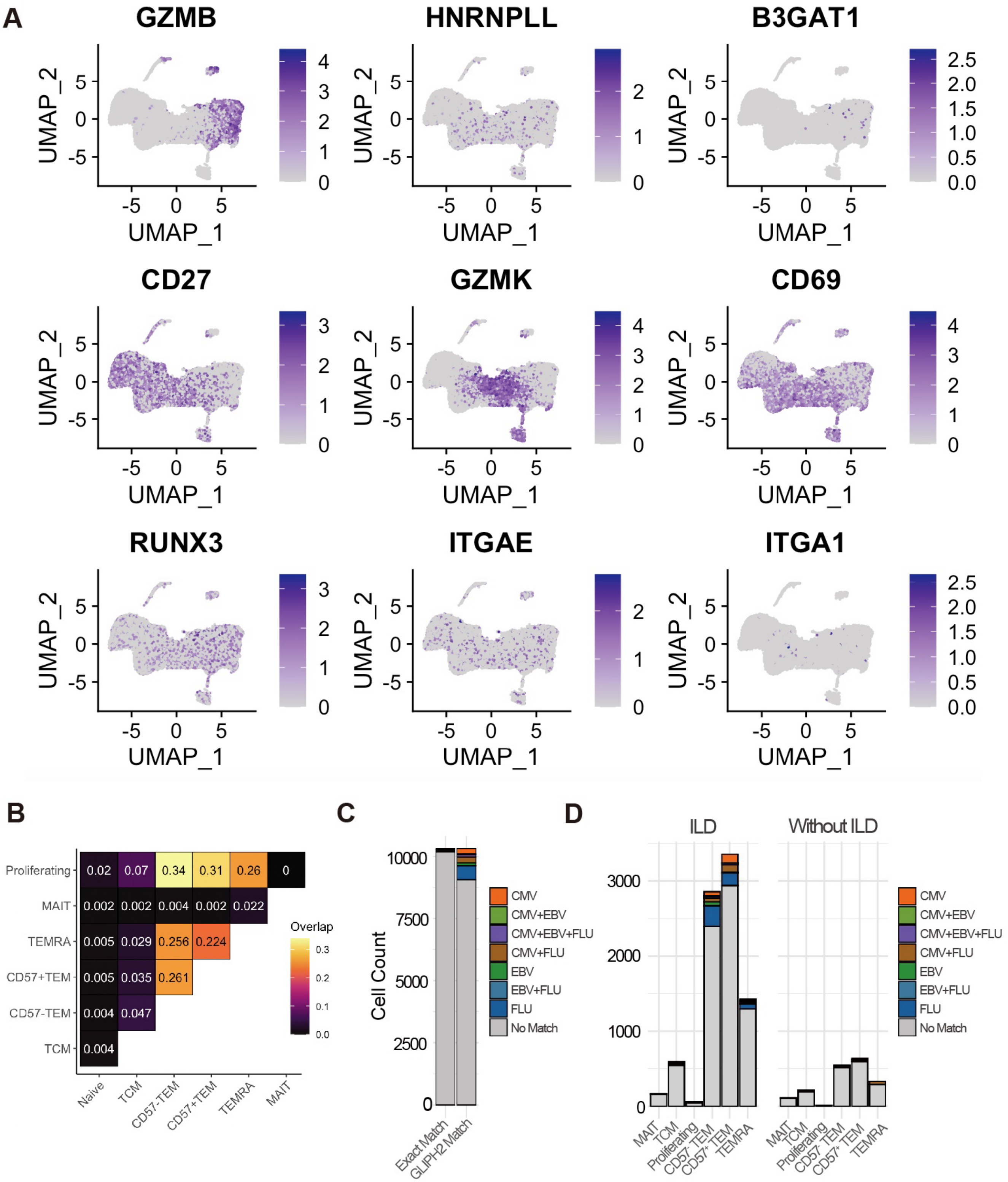
A
GZMB
HNRNPLL
B3GAT1
CD27
GZMK
CD69
RUNX3
ITGAE
ITGA1
UMAP_1
UMAP_2
B
Proliferating
MAIT
TEMRA
CD57+TEM
CD57-TEM
TCM
Naive
0.02 0.07 0.34 0.31 0.26 0
0.002 0.002 0.004 0.002 0.022
0.005 0.029 0.256 0.224
0.005 0.035 0.261
0.004 0.047
0.004
Overlap
C
Cell Count
Exact Match
GLIPH2 Match
D
ILD
Without ILD
MAIT
TCM
Proliferating
CD57- TEM
CD57+ TEM
TEMRA
CMV
CMV+EBV
CMV+EBV+FLU
CMV+FLU
EBV
EBV+FLU
FLU
No Match

**Supplemental Figure 3. Gene expression of CD57$^+$ TEM in scRNAseq data**

**A.** Gene expression of *GZMB*, *HNRNPLL*, *B3GAT1*, *CD27*, *GZMK*, *CD69*, *RUNX3*, *ITGAE*, and *ITGA1* in scRNA-seq dataset from PBMC. **B.** TCR clonal overlap across indicated CD8 T cell clusters. **C.** Bar graphs of cell counts stratified by viral reactive TCRs match to known viral TCRs or inferred by GLIPH2 motif analysis. **D.** Predicted viral reactivity of cells using GLIPH2 of expanded CD8 subsets comparing between SSc-ILD and SSc without ILD. Bars stratified by predicted viral reactivity.

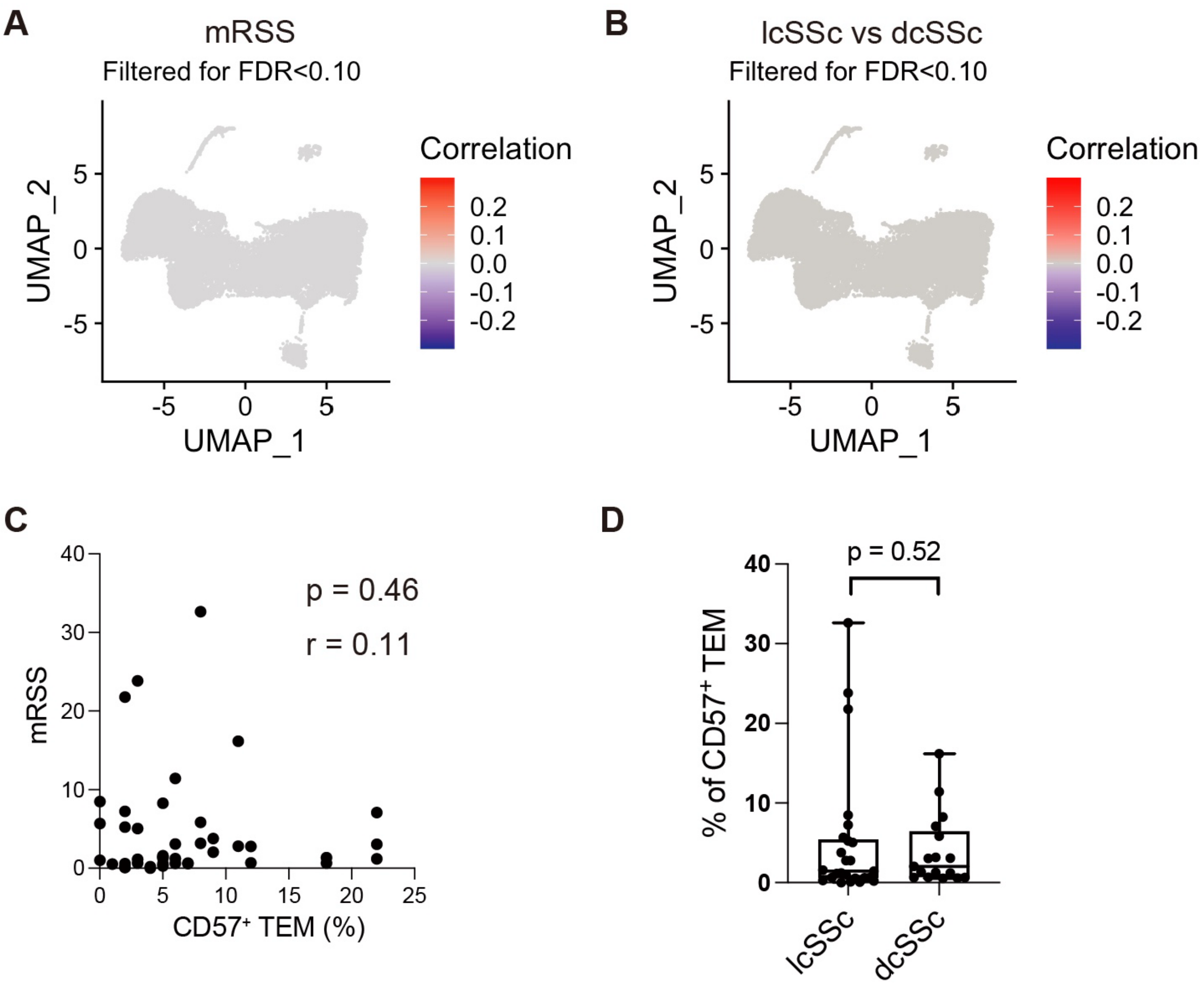


**Supplemental Figure 4. No association between CD57$^+$ TEM and skin involvement.**

**A, B.** CNA to evaluate the association between CD8 T cell cluster and severity of skin involvement, adjusting for age and sex (Cohort 1). **C.** Correlation between mRSS and CD57$^+$ TEM (Cohort 2). **D.** Comparison of CD57+TEM between lcSSc and dcSSc. Mann-Whitney U test.

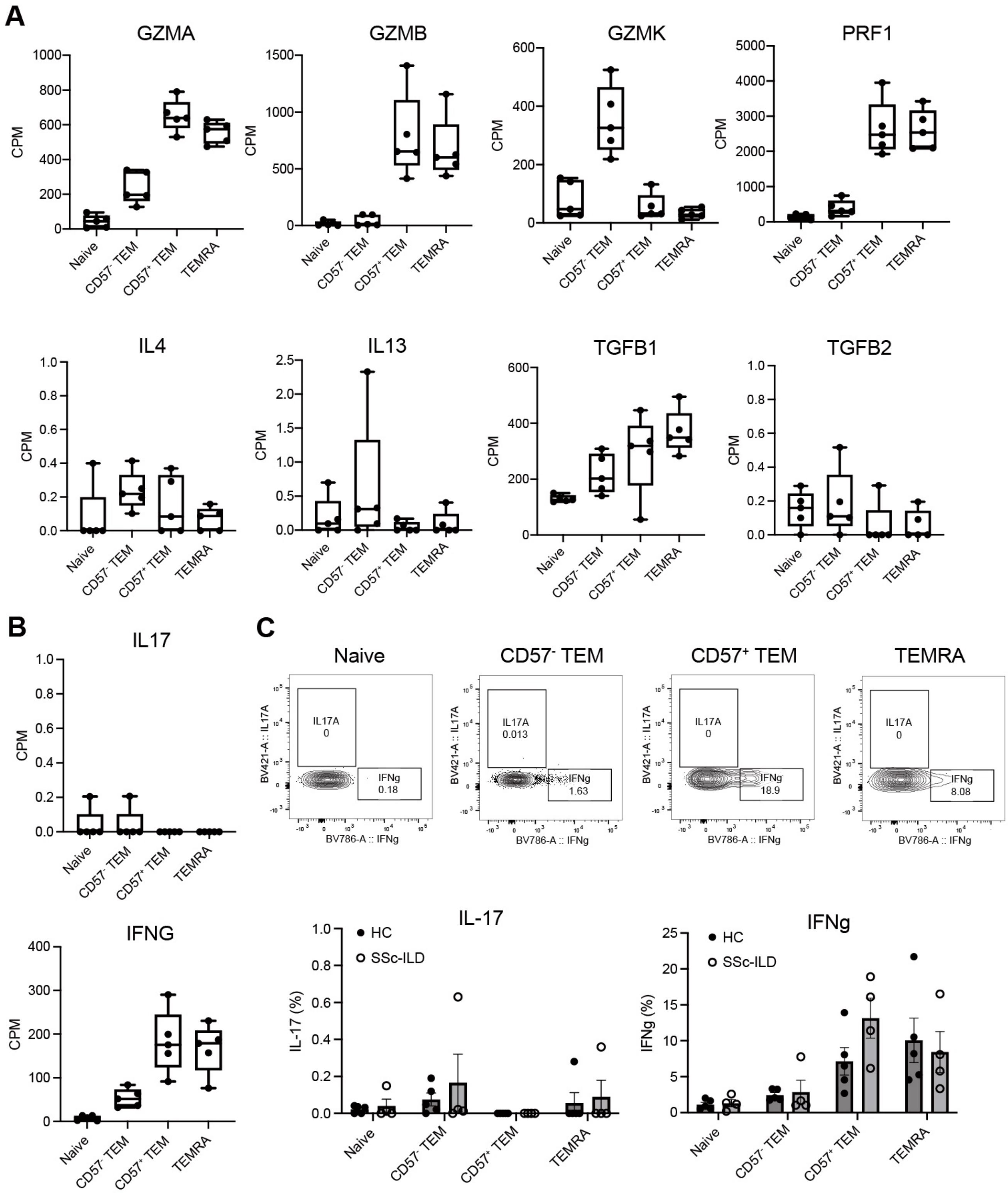
A
GZMA
GZMB
GZMK
PRF1
IL4
IL13
TGFB1
TGFB2
CPM
Naive
CD57⁻ TEM
CD57⁺ TEM
TEMRA
B
IL17
IFNG
C
Naive
CD57⁻ TEM
CD57⁺ TEM
TEMRA
BV421-A :: IL17A
BV786-A :: IFNg
IL17A 0
IFNg 0.18
IL17A 0.013
IFNg 1.63
IL17A 0
IFNg 18.9
IL17A 0
IFNg 8.08
IL-17
IL-17 (%)
IFNg
IFNg (%)
HC
SSc-ILD

**Supplemental Figure 5. Gene expression in the bulk RNA-seq.**

**A, B.** Gene expression of *GZMA*, *GZMB*, *GZMK*, *PRF1*, *IL4*, *IL13*, *TGFB1*, *TGFB2, IL17*, and *IFNG* in naive, $CD57^-$ TEM, $CD57^+$ TEM, and TEMRA CD8 T cells sorted from 5 SSc-ILD donors. CPM: Counts per million. **C.** Intracellular staining of IL-17 and IFNg in naive, $CD57^-$ TEM, $CD57^+$ TEM, and TEMRA CD8 T cells stimulated with anti-CD3 antibody (SK7, 5ug/ml) for 5 hours. HC: n = 5, SSc-ILD: n = 4.

A

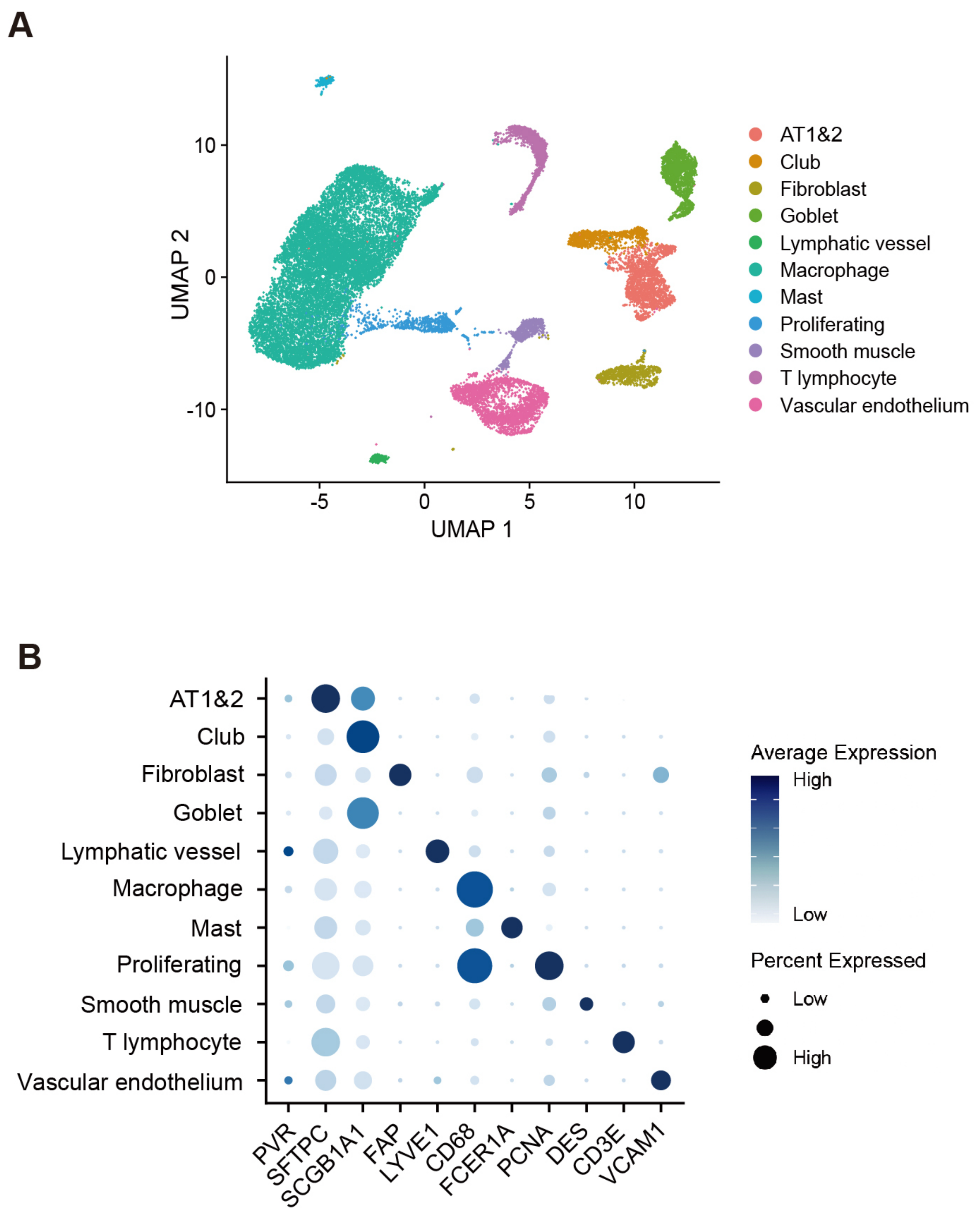

A
UMAP 2
UMAP 1
10
0
-10
-5
0
5
10
AT1&2
Club
Fibroblast
Goblet
Lymphatic vessel
Macrophage
Mast
Proliferating
Smooth muscle
T lymphocyte
Vascular endothelium
B
AT1&2
Club
Fibroblast
Goblet
Lymphatic vessel
Macrophage
Mast
Proliferating
Smooth muscle
T lymphocyte
Vascular endothelium
PVR
SFTPC
SCGB1A1
FAP
LYVE1
CD68
FCER1A
PCNA
DES
CD3E
VCAM1
Average Expression
High
Low
Percent Expressed
Low
High

**Supplemental Figure 6. CD155/PVR expression in lung tissues from patients with SSc-ILD.**

**A.** UMAP clustering of all cells in scRNA-seq from lung samples of 4 SSc-ILD donors (*24*). **B.** *CD155/PVR* expression in clusters from scRNA-seq data.

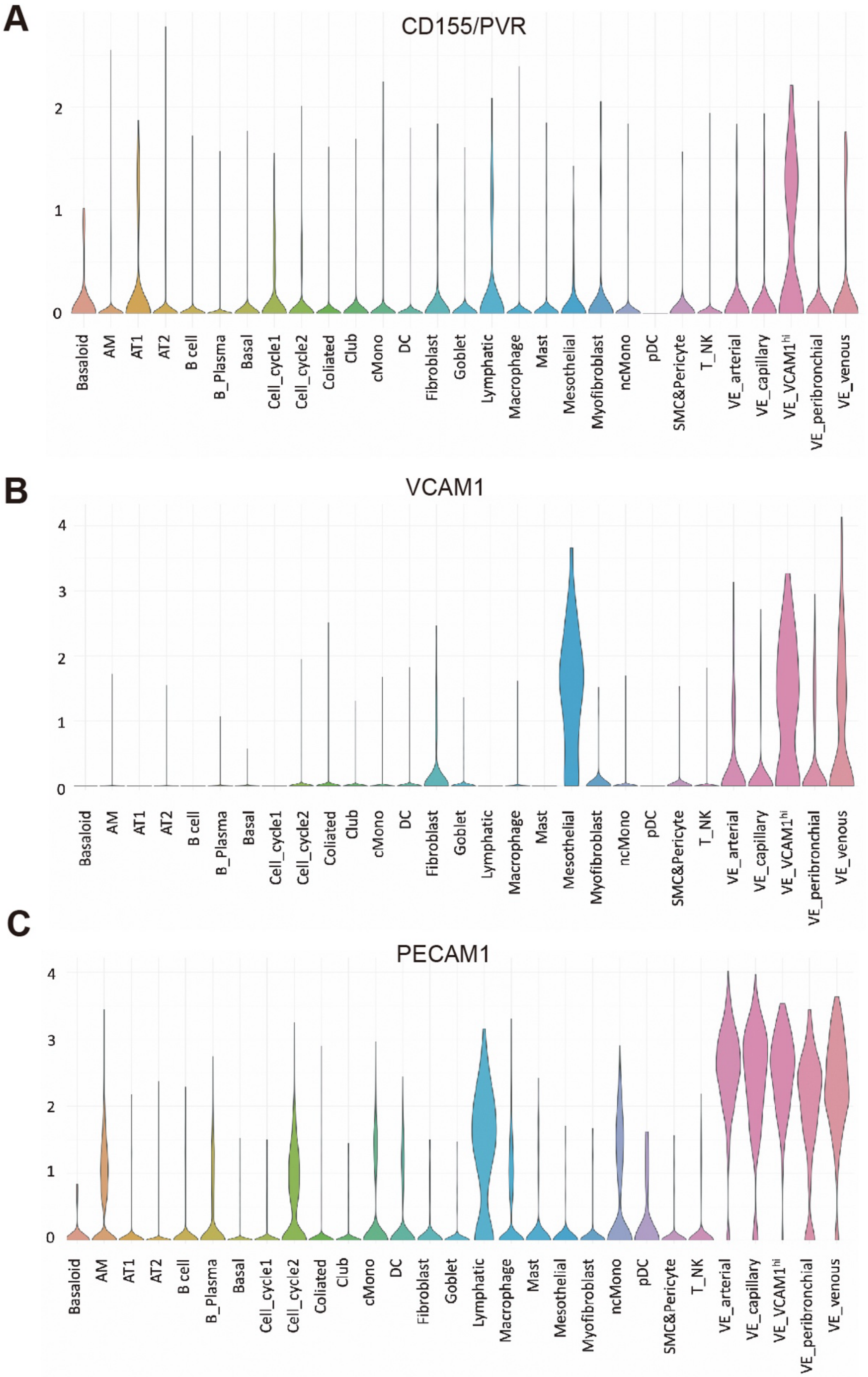
A
CD155/PVR
0
1
2
Basaloid
AM
AT1
AT2
B cell
B_Plasma
Basal
Cell_cycle1
Cell_cycle2
Coliated
Club
cMono
DC
Fibroblast
Goblet
Lymphatic
Macrophage
Mast
Mesothelial
Myofibroblast
ncMono
pDC
SMC&Pericyte
T_NK
VE_arterial
VE_capillary
VE_VCAM1hi
VE_peribronchial
VE_venous
B
VCAM1
0
1
2
3
4
C
PECAM1
0
1
2
3
4

**Supplemental Figure 7. CD155/PVR expression in lung tissues from IPF Cell Atlas.**

**A-C.** *CD155/PVR*, *VCAM1*, and *PECAM1* expression in clusters from the IPF Cell Atlas scRNA-seq data (*34*, *35*).

**Supplemental Table 1. DEGs (FDR < 0.1) that are highly upregulated in CD57$^+$ TEM compared to TEMRA (bulk RNA-seq)**

| Gene | Log2 fold change | P value | Adjusted P value |
|---|---|---|---|
| HNRNPLL | 1.73607579 | 1.0586908553705e-11 | 3.59610816297974e-08 |
| GALM | 1.21854429 | 1.15847120649999e-07 | 0.00010493 |
| ANXA5 | 0.76757603 | 1.32287624641183e-06 | 0.00084321 |
| PDE4B | 0.85726532 | 1.92667886051448e-06 | 0.00113817 |
| CD5 | 0.72223552 | 4.44535011243582e-06 | 0.00232304 |
| ITGB1 | 0.74006799 | 9.32026618114284e-06 | 0.00410469 |
| CD40LG | 2.78512365 | 2.26585201442019e-05 | 0.00879604 |
| CD52 | 0.61556933 | 2.96683212642584e-05 | 0.01033599 |
| CD70 | 1.3129868 | 3.30615229792123e-05 | 0.01123017 |
| S100A4 | 0.42527575 | 3.86305904430508e-05 | 0.012497 |
| MAST4 | 0.90812319 | 4.16673197513938e-05 | 0.0131659 |
| ITGA1 | 3.15188845 | 5.4743663562247e-05 | 0.01616961 |
| NSG1 | 0.98918524 | 6.04688922900141e-05 | 0.01711648 |
| UBXN11 | 0.54123019 | 7.32750474714714e-05 | 0.01878468 |
| SH3RF2 | 2.85023009 | 7.4755907616438e-05 | 0.01880942 |
| ITGA4 | 0.41998442 | 8.06953318498633e-05 | 0.01993468 |
| ALOX5AP | 0.73735714 | 8.66311764179525e-05 | 0.02101889 |
| CD2 | 0.62368285 | 0.00011393 | 0.02623563 |
| RN7SL1 | 0.7932057 | 0.00013893 | 0.03044626 |
| LTB | 0.76046986 | 0.00014552 | 0.03138376 |
| SLC43A3 | 0.64879621 | 0.00015291 | 0.03205593 |
| OASL | 0.66993897 | 0.0001703 | 0.03481681 |
| LGALS3BP | 1.43774483 | 0.00021795 | 0.04134665 |
| MAP3K13 | 0.7770777 | 0.0002191 | 0.04134665 |
| LOC101928034 | 0.39031938 | 0.00033733 | 0.05658419 |
| ICOS | 0.71705771 | 0.00035055 | 0.05808443 |
| CERKL | 0.44821971 | 0.00038172 | 0.06030766 |

| | | | |
|---|---|---|---|
| SSBP2 | 0.91456277 | 0.00041685 | 0.06435998 |
| GADD45G | 0.53037385 | 0.00044336 | 0.06693246 |
| FGFBP2 | 0.47572233 | 0.00045591 | 0.06791033 |
| S100A11 | 0.4596911 | 0.00049076 | 0.07093578 |
| ITM2C | 1.35589093 | 0.00054188 | 0.07750081 |
| EPHX4 | 1.30086204 | 0.0005627 | 0.07963961 |
| CTLA4 | 1.85502062 | 0.00067746 | 0.09126739 |
| KCNN4 | 0.94681148 | 0.00069699 | 0.09204558 |
| SNORA104 | 0.73931104 | 0.00070173 | 0.09204558 |
| TMEM173 | 0.35057535 | 0.00071238 | 0.09218244 |
| E2F2 | 1.09675748 | 0.00074735 | 0.09254052 |
| APOBEC3H | 0.71960803 | 0.00074921 | 0.09254052 |
| PHLDA1 | 0.60762598 | 0.00074268 | 0.09254052 |
| DOK6 | 1.90952276 | 0.00075845 | 0.09283849 |
| HDAC11 | 0.62063378 | 0.00084009 | 0.09947116 |
| PLOD3 | 0.47496495 | 0.00084176 | 0.09947116 |
| PRDX2 | 0.3987129 | 0.00084192 | 0.09947116 |

**Supplemental Table 2. DEGs (FDR < 0.1) that are highly upregulated in TEMRA compared to CD57$^+$ TEM (bulk RNA-seq)**

| Gene | Log2 fold change | P value | Adjusted P value |
|---|---|---|---|
| GCSAM | 3.13391165 | 2.47572002840619e-16 | 3.3637608025955e-12 |
| CXXC5 | 2.28024627 | 2.07877364020776e-13 | 1.41221487247514e-09 |
| NCR1 | 2.33880965 | 4.56917771802856e-13 | 2.06938058849514e-09 |
| IRF8 | 2.24167179 | 2.69076824267969e-11 | 7.3118936226578e-08 |
| KIFC3 | 1.55714794 | 4.36510605972781e-11 | 9.88478267225362e-08 |
| CNR2 | 2.22479245 | 4.98790937619831e-10 | 9.68153209920091e-07 |
| CD160 | 1.43834208 | 4.18147497397073e-09 | 7.10171255891753e-06 |
| CTBP2 | 1.72045803 | 1.30380312413103e-08 | 1.96830811639648e-05 |
| IKZF2 | 4.91261302 | 2.61962905530961e-08 | 3.55928999744917e-05 |
| PDE4A | 1.51003806 | 2.92049057952012e-08 | 3.60733686399453e-05 |
| KLRC2 | 2.87874606 | 3.33493422657442e-08 | 3.77597927803888e-05 |
| PIK3AP1 | 1.38363362 | 4.53555325878861e-08 | 4.74035093285852e-05 |
| HIPK2 | 1.17234671 | 7.59686793347185e-08 | 7.37276032943443e-05 |
| TKTL1 | 2.1149194 | 2.23163175855082e-07 | 0.00018951 |
| GFOD1 | 1.31879151 | 2.55511592334906e-07 | 0.00020421 |
| SPRY2 | 3.86244472 | 3.0543655521328e-07 | 0.00023055 |
| MYRF | 1.30940391 | 1.03230900181051e-06 | 0.00073821 |
| CHST2 | 2.28265239 | 1.20495500889468e-06 | 0.00081859 |
| NLRP7 | 2.63936375 | 1.36532238429179e-06 | 0.00084321 |
| CMC1 | 0.68643105 | 2.34914767695185e-06 | 0.00132991 |
| FEZ1 | 1.15250983 | 3.26984355669285e-06 | 0.0017771 |
| TOR4A | 2.15845146 | 7.69306042456997e-06 | 0.00373711 |
| KLRC3 | 2.68093069 | 7.70141941329079e-06 | 0.00373711 |
| LYN | 1.40609385 | 9.4229047711926e-06 | 0.00410469 |
| TYROBP | 2.66157334 | 9.66732834869963e-06 | 0.00410469 |
| TXK | 3.33374523 | 9.38146866115594e-06 | 0.00410469 |
| PRSS21 | 1.71635129 | 1.47103165938524e-05 | 0.00605664 |

| | | | |
|---|---|---|---|
| PITPNM2 | 1.71046616 | 2.14500697645504e-05 | 0.00857183 |
| C17ORF107 | 1.07423264 | 2.78906733359053e-05 | 0.01033599 |
| PDLIM7 | 1.19917686 | 2.93622054573985e-05 | 0.01033599 |
| KLRF1 | 2.04864389 | 2.96158235511361e-05 | 0.01033599 |
| HDAC7 | 0.81969386 | 3.49539440015207e-05 | 0.0115834 |
| RIN3 | 0.8516555 | 5.22593551680496e-05 | 0.01613745 |
| RHOBTB3 | 1.81642478 | 5.4712687711234e-05 | 0.01616961 |
| TSPOAP1 | 0.74720844 | 5.62444349289645e-05 | 0.01625943 |
| ARAP3 | 1.56545517 | 6.59247266091775e-05 | 0.01827999 |
| CALHM6 | 1.65250854 | 6.78207796004806e-05 | 0.01842962 |
| TJP3 | 1.30684264 | 7.00403551485291e-05 | 0.01865958 |
| LAT2 | 2.82282388 | 7.18548400716816e-05 | 0.01877484 |
| HMOX1 | 3.95983299 | 9.50212892181926e-05 | 0.02265008 |
| SLC4A4 | 0.97816185 | 0.00010806 | 0.02531336 |
| LYST | 0.51093721 | 0.00012887 | 0.0291836 |
| APBA2 | 0.77458131 | 0.00013187 | 0.02937151 |
| TCF7 | 0.85433633 | 0.00015336 | 0.03205593 |
| RFTN1 | 0.6145366 | 0.00017169 | 0.03481681 |
| SIGLEC7 | 2.36318501 | 0.00018637 | 0.03723925 |
| CERS4 | 0.9688773 | 0.00021805 | 0.04134665 |
| ARVCF | 1.42465719 | 0.00021783 | 0.04134665 |
| ZNF320 | 1.25782872 | 0.00022754 | 0.04234962 |
| PLCL1 | 1.29330474 | 0.00025592 | 0.04698957 |
| VIPR2 | 1.64461287 | 0.00026155 | 0.04738303 |
| ZNF853 | 1.38220646 | 0.00026724 | 0.0475384 |
| OTOF | 1.55420329 | 0.00026941 | 0.0475384 |
| FCMR | 0.50438761 | 0.00028428 | 0.04951941 |
| KLHL4 | 4.64574804 | 0.00030067 | 0.0517122 |
| TLE1 | 2.24056698 | 0.00031498 | 0.05349611 |
| PLAC8 | 0.55310125 | 0.00036729 | 0.05940899 |
| BOK | 2.54764884 | 0.00036679 | 0.05940899 |

| | | | |
|---|---|---|---|
| FCGR3A | 0.6302477 | 0.00037481 | 0.05991298 |
| WHAMMP2 | 1.3259953 | 0.00040139 | 0.06268531 |
| TRPV3 | 1.92755557 | 0.00043461 | 0.06634802 |
| ERGIC1 | 0.86894663 | 0.00046225 | 0.06791033 |
| QSER1 | 1.17357231 | 0.00046483 | 0.06791033 |
| ACTN1 | 0.92707048 | 0.00058994 | 0.08263409 |
| PLCG2 | 0.86847902 | 0.0006463 | 0.08943343 |
| MAFF | 1.1633763 | 0.00065165 | 0.08943343 |
| CHN2 | 0.89596163 | 0.00067844 | 0.09126739 |
| LIMK1 | 0.71759156 | 0.00070455 | 0.09204558 |
| PRR7 | 0.86430757 | 0.00072667 | 0.09254052 |
| WHAMMP3 | 1.13019886 | 0.00073217 | 0.09254052 |
| VANGL1 | 0.79510302 | 0.00076637 | 0.09296998 |

**Supplemental Table 3. Top 50 DEGs (FDR < 0.1) that are highly upregulated in CD57$^{+}$ TEM compared to TEMRA (scRNA-seq)**

| Gene | Log2 fold change | P value | Adjusted P value |
|---|---|---|---|
| S100A4 | 0.75198384 | 8.58482678181056e-299 | 2.87917920608363e-294 |
| CD52 | 0.57096088 | 6.3648822845047e-217 | 2.13465422057719e-212 |
| GNLY | 0.64994509 | 5.22171212629676e-140 | 1.75125781291741e-135 |
| FGFBP2 | 0.68342811 | 2.6267338609579e-132 | 8.8095400228806e-128 |
| LGALS1 | 0.85502326 | 1.35142657745298e-127 | 4.53241445546179e-123 |
| S100A6 | 0.50004056 | 2.72529485428746e-122 | 9.14009388230929e-118 |
| ZNF683 | 1.19927629 | 1.17157128498506e-110 | 3.92921577558288e-106 |
| GZMH | 0.42201448 | 3.69728239458519e-87 | 1.23999456949598e-82 |
| ITGB1 | 0.85394504 | 2.87035797160349e-85 | 9.62660656516378e-81 |
| CD8A | 0.30699539 | 1.72963606388147e-69 | 5.80085343104568e-65 |
| TMSB10 | 0.21292985 | 1.98886642816712e-66 | 6.6702602267869e-62 |
| SH3BGRL3 | 0.26688593 | 1.93017950996152e-63 | 6.47343604050894e-59 |
| S100A10 | 0.43352995 | 8.56161249350115e-61 | 2.87139359807042e-56 |
| PROK2 | 2.60932513 | 1.703282540737e-58 | 5.71246898512374e-54 |
| RPS27 | 0.16711494 | 4.77525536445499e-54 | 1.60152514413091e-49 |
| RPL26 | 0.16665551 | 3.72822042387187e-53 | 1.25037056575815e-48 |
| CD6 | 0.79388774 | 1.06633953041962e-49 | 3.57628951712131e-45 |
| MALAT1 | 0.43436619 | 1.00692494509465e-47 | 3.37702488085845e-43 |
| CD2 | 0.53019172 | 2.20903388301016e-46 | 7.40865783683949e-42 |
| RPL39 | 0.1779919 | 1.1202123847817e-45 | 3.75696829608086e-41 |
| RPS4X | 0.16008027 | 1.49234679667864e-44 | 5.00503268670083e-40 |
| THEMIS | 0.95040129 | 4.88051310587496e-44 | 1.63682648544834e-39 |
| RPS24 | 0.17867761 | 1.48765853995687e-43 | 4.98930921130735e-39 |
| GZMB | 0.35196553 | 6.04387739904136e-41 | 2.02699560209049e-36 |
| PRSS23 | 0.69238262 | 9.11728351652073e-40 | 3.05775454577072e-35 |
| TRBV27 | 1.78900695 | 3.53229093541691e-38 | 1.18465973392012e-33 |
| MT-CYB | 0.22401699 | 8.5389151145372e-38 | 2.86378135111349e-33 |

| | | | |
|---|---|---|---|
| CD8B | 0.45967639 | 1.62291970615917e-37 | 5.44294811051663e-33 |
| RPL34 | 0.14685022 | 2.09762299702087e-37 | 7.0350080074086e-33 |
| TRAV8-1 | 2.6325029 | 4.8959037709206e-36 | 1.64198820669135e-31 |
| RPS14 | 0.13534851 | 2.84582035925105e-35 | 9.54431232085618e-31 |
| AHNAK | 0.42201348 | 3.59313379215876e-33 | 1.20506521121421e-28 |
| RPS27A | 0.12454844 | 8.51879440574673e-33 | 2.85703326779934e-28 |
| ANXA1 | 0.32252258 | 1.05218079358654e-32 | 3.52880394553055e-28 |
| MT-ND5 | 0.24175171 | 1.54245682194063e-32 | 5.17309168942449e-28 |
| CD226 | 1.06242906 | 1.30580667423439e-31 | 4.37941442404729e-27 |
| CD5 | 0.7150902 | 4.42287612850442e-31 | 1.48334419597781e-26 |
| VIM | 0.33616994 | 7.53557016410622e-31 | 2.52727952163794e-26 |
| TRBV10-3 | 2.1435419 | 7.5694015895595e-30 | 2.53862590510646e-25 |
| MYOM2 | 1.60832371 | 4.01865410586711e-29 | 1.34777621402571e-24 |
| ANXA5 | 0.63625063 | 6.88920703000034e-28 | 2.31050225372152e-23 |
| RPL7A | 0.12885182 | 4.62649856515772e-27 | 1.5516350887826e-22 |
| RPS15 | 0.12424821 | 1.00942400936203e-25 | 3.38540624259837e-21 |
| RPS21 | 0.15627078 | 1.34178108326093e-25 | 4.5000653970405e-21 |
| CES1 | 1.25725772 | 1.45594709552273e-25 | 4.88295536896413e-21 |
| FLNA | 0.33446993 | 3.77627867209507e-25 | 1.26648834104725e-20 |
| MT-ND3 | 0.22006776 | 1.72944781369369e-24 | 5.80022207756588e-20 |
| RPS3 | 0.10613317 | 3.30879372272311e-23 | 1.10970323872688e-18 |
| RPL10 | 0.10093032 | 4.9854865828242e-22 | 1.67203249014758e-17 |
| TSPAN2 | 0.99868105 | 9.05550617256172e-22 | 3.03703566015375e-17 |

**Supplemental Table 4. Top 50 DEGs (FDR < 0.1) that are highly upregulated in TEMRA compared to CD57$^+$ TEM (scRNA-seq)**

| Gene | Log2 fold change | P value | Adjusted P value |
|---|---:|---|---|
| IFI44L | 3.55034086 | 8.71530680694944e-235 | 2.9229395969147e-230 |
| MX1 | 2.43583869 | 4.34155614236847e-169 | 1.45607109902754e-164 |
| TYROBP | 2.4853734 | 2.42775982835871e-164 | 8.14222091234945e-160 |
| KLRF1 | 2.55598683 | 1.35349024483816e-149 | 4.53933558313821e-145 |
| CMC1 | 1.4185647 | 9.47573478544335e-141 | 3.17797193234199e-136 |
| IFI6 | 1.82488593 | 4.61775304262872e-133 | 1.54870201543682e-128 |
| TRDC | 4.72524498 | 2.13964195432368e-132 | 7.17593118641077e-128 |
| KLRC3 | 2.49004235 | 1.85917352858029e-128 | 6.23529618015258e-124 |
| XAF1 | 1.48914744 | 6.42045373114354e-127 | 2.15329177235092e-122 |
| TIGIT | 1.6841964 | 9.17327136636238e-124 | 3.07653175085061e-119 |
| IKZF2 | 3.74363986 | 1.21196185851481e-121 | 4.06467768108696e-117 |
| GZMK | 2.24760924 | 6.09695941423323e-120 | 2.04479824834554e-115 |
| DUSP2 | 1.09369207 | 1.24013523829512e-116 | 4.15916556219417e-112 |
| OAS1 | 2.11062952 | 1.18026174557379e-105 | 3.95836184230539e-101 |
| CD7 | 0.77717422 | 4.31814475745142e-105 | 1.44821938875406e-100 |
| ISG15 | 1.44218842 | 6.1712435325767e-104 | 2.06971165595557e-99 |
| IFITM1 | 0.50280702 | 1.45774342853471e-103 | 4.88897991061972e-99 |
| TRDV1 | 5.02508843 | 4.97837396734879e-103 | 1.66964706116944e-98 |
| IFI27 | 3.99616487 | 3.06340170053963e-97 | 1.02740366232698e-92 |
| USP18 | 3.15147839 | 3.8405715366914e-93 | 1.28805088197556e-88 |
| CD27 | 1.88867813 | 2.73837585283501e-91 | 9.18396493523806e-87 |
| IRF7 | 1.5070033 | 2.04102972707301e-88 | 6.84520549865746e-84 |
| IFITM3 | 1.48249613 | 2.52390061003972e-82 | 8.46465786595121e-78 |
| KIR3DL2 | 2.86544284 | 3.16594088049851e-82 | 1.06179325250159e-77 |
| KLRB1 | 1.25117323 | 9.21045290563691e-82 | 3.08900169549251e-77 |
| LGALS9 | 2.12545977 | 1.04018009488324e-81 | 3.48855600221943e-77 |
| SPARC | 3.32616791 | 1.12916198914898e-81 | 3.78698347920784e-77 |

| | | | |
|---|---|---|---|
| LYST | 1.29296813 | 6.44827214671833e-81 | 2.16262151256639e-76 |
| PLSCR1 | 1.78102752 | 1.99152462507816e-79 | 6.67917528758712e-75 |
| TUBB1 | 3.50330353 | 8.23503421967689e-77 | 2.76186577659523e-72 |
| DUSP1 | 0.78319068 | 1.58336488266618e-76 | 5.31028914348584e-72 |
| ZFP36 | 0.73278748 | 4.02311249172819e-75 | 1.3492714674758e-70 |
| CD69 | 0.87883577 | 1.4253598964694e-74 | 4.78037202077906e-70 |
| PPBP | 2.58161763 | 1.96369732656708e-74 | 6.58584809384066e-70 |
| ISG20 | 0.79412815 | 2.22001532891283e-73 | 7.44548741010786e-69 |
| PLAC8 | 0.95777237 | 2.66125161099379e-72 | 8.92530565295097e-68 |
| NCR1 | 2.35750256 | 1.93305047662802e-71 | 6.48306468851505e-67 |
| EPSTI1 | 1.45449217 | 4.89334836277195e-71 | 1.64113117390646e-66 |
| FCMR | 0.90710838 | 5.19501139938105e-71 | 1.74230292312442e-66 |
| GCSAM | 4.88130419 | 8.1119854525235e-71 | 2.72059768106733e-66 |
| IFI44 | 2.12561788 | 1.32138130985072e-69 | 4.43164863697736e-65 |
| TRBV7-9 | 1.77508152 | 2.96579511338606e-68 | 9.94668365127417e-64 |
| GNG11 | 2.85515496 | 7.95554773563869e-68 | 2.6681315995785e-63 |
| RSAD2 | 2.909819 | 2.69019112534093e-67 | 9.02236299616842e-63 |
| CAVIN2 | 2.76025515 | 2.29539740956783e-64 | 7.69830383220858e-60 |
| KIR2DL3 | 3.27249774 | 4.43563961118888e-63 | 1.48762481280053e-58 |
| NRGN | 2.54711482 | 5.53881799041518e-61 | 1.85760877762544e-56 |
| CD74 | 0.41345717 | 1.49712486788258e-59 | 5.02105738190459e-55 |
| EIF2AK2 | 1.1590259 | 4.93468867100934e-58 | 1.65499588648311e-53 |
| CD160 | 1.43505424 | 4.77021769635604e-56 | 1.59983561100389e-51 |

**Supplemental Table 5. scRNAseq sample information.**

| | Number of batches | 3' or 5' | TCR data | Total number of cells detected | Number of CD8 T cells detected | Number of donors | Number of ILD donors |
|---|---|---|---|---|---|---|---|
| | 2 | 3' prime | No | 146,531 | 1,569 | 10 | 7 |
| | 4 | 5' prime | Yes | 204,950 | 27,593 | 50 | 33 |
| Total | 6 | | | 351,481 | 29,162 | 58* | 38* |

* The data from 2 donors were generated by both Chromium Next GEM Single Cell 3' Kit and Chromium Next GEM Single Cell 5' Kit.

**Supplemental Table 6. List of flow cytometry antibodies.**

| Antigen | Color | Manufacturer | Product # |
|---|---|---|---|
| CD3 | AF700 | BioLegend | 300424 |
| CD8 | APC-Cy7 | BioLegend | 344714 |
| CD56 | BV510 | BioLegend | 362534 |
| CD57 | FITC | BioLegend | 359603 |
| CD27 | PE-Cy7 | BioLegend | 356412 |
| CD45RA | BV650 | BioLegend | 304135 |
| CCR7 | PerCP/Cyanine5.5 | BioLegend | 353220 |
| TIGIT | PE | BioLegend | 372704 |
| CD226 | APC | BioLegend | 338311 |
| CD49d | BV711 | BioLegend | 304322 |
| CD29 | BV421 | BD | 743783 |
| Zombi UV | AF350 | BioLegend | 423108 |
| CD3 | APC-Cy7 | BioLegend | 344818 |
| CD8 | BV605 | BioLegend | 344742 |
| CD57 | AF647 | BioLegend | 359614 |
| CD45RA | PE-Cy7 | BioLegend | 304126 |
| CD27 | BV421 | BioLegend | 356412 |
| GZMK | FITC | BioLegend | 370508 |
| GZMB | AF700 | BioLegend | 372221 |

**Supplemental Table 7**. **List of mass cytometry antibodies.**

| Antigen | Clone | Metal |
|---|---|---|
| CD20 | 2H7 | 113In |
| CD3 | UCHT1 | 115In |
| CCR6 | G034E3 | 141Pr |
| CD57 | HCD57 | 142Nd |
| CD123 | 6H6 | 143Nd |
| GraB | GB11 | 144Nd |
| CRTH2 | BM16 | 145Nd |
| CD8a | RPA T8 | 146Nd |
| CD45RO | UCHL1 | 147Sm |
| CD28 | CD28.2 | 148Nd |
| Grak | GM26E7 | 149Sm |
| CD11c | Bu15 | 150Nd |
| ICOS | C398.4A | 151Eu |
| CD14 | M5E2 | 152Sm |
| CCR2 | K036C2 | 153Eu |
| TIGIT | MBSA43 | 154Sm |
| CD4 | RPA T4 | 155Gd |
| CD73 | AD2 | 156Gd |
| CD16 | 3G8 | 157Gd |
| CD27 | L128 | 158Gd |
| CCR7 | G043H7 | 159Tb |
| CXCR6 | K041E5 | 160Gd |
| T-bet | 4B10 | 161Dy |
| FoxP3 | PCH101 | 162Dy |
| CXCR3 | G025H7 | 163Dy |
| CXCR5 | J252D4 | 164Dy |
| CD127 | TX31 | 165Ho |
| GATA3 | TWAJ | 166Er |
| CD38 | HTA125 | 167Er |
| CCR9 | L053E8 | 168Er |
| TCRgd | B1 | 169Tm |
| CX3CR1 | 2A9-1 | 170Er |
| CCR5 | NP6G4 | 171Yb |
| Ki67 | B56 | 172Yb |
| CD25 | M-A251 | 173Yb |
| CCR10 | 6588-5 | 174Yb |
| PD-1 | EH12.2H7 | 175Lu |
| CD56 | R19760 | 176Yb |
| HLA-DR | BVD2-23B6 | 209Bi |
